# Representing national images of self and others through China’s diplomatic discourse: A corpus-based study

**DOI:** 10.1371/journal.pone.0350726

**Published:** 2026-06-12

**Authors:** Feng Pan, Weixiao Li, Tao Li

**Affiliations:** 1 Institute of Translation Studies, Shanghai International Studies University, Shanghai, China; 2 German School Shanghai, Shanghai, China; 3 College of Foreign Languages, Shanghai Maritime University, Shanghai, China; NingboTech University, CHINA

## Abstract

Drawing on van Dijk’s Ideological Square framework, this paper adopts a corpus-based method to examine the discursive strategies in their responses by the spokespersons for China’s Ministry of Foreign Affairs during regular press conferences amid a public health crisis. The analysis focuses on how these discursive strategies shape the national images of China and the other four permanent members of the United Nations Security Council. The results show that (1) the spokespersons actively employed communicative discursive strategy to clarify China’s stance and international cooperation initiatives while also using offensive discourse strategy to counter criticisms from US-led Western nations and media regarding the virus and the pandemic; (2) although the spokespersons’ discourse generally aligns with van Dijk’s Ideological Square of positive self-presentation and negative other-presentation, this model is not fixed but subject to dynamic changes driven by the self-serving principle. It is argued that factors such as diplomatic ideology, geopolitical relations, and traditional Chinese culture underlie the spokespersons’ use of discursive strategies and national images representations. This study contributes to reconceptualizing an existing discourse model by offering data-driven insights into the operational mechanisms of ideological discourse in the contexts of global political communication and national image construction.

## Introduction

Within international diplomatic studies, discourse in diplomatic settings has increasingly been recognized as a key site where national interests, diplomatic ideology, and power relations are discursively negotiated, particularly in times of international crisis [1–5]. The discursive features of the spokespersons for a country’s Ministry of Foreign Affairs have particularly raised great attention as they use linguistic and communicative skills to convey their country’s attitudes, explain policies, and address international doubts and concerns [6–11]. In the context of public health crises, the discursive controversy surrounding COVID-19 between China and the US-led Western countries evolved into a contest for international discursive power and has led to extensive discussions [12–19]. Such discussions have also been reflected in diplomatic discourse, particularly those produced by the spokespersons for a country’s Ministry of Foreign Affairs.

Regarding the features of the spokespersons’ discourse, Wu [20] examined the discursive style of the spokespersons for the Ministry of Foreign Affairs of China (MFAC), finding that the spokespersons often employ a confrontational style in their responses to target critics of China regarding COVID-19 or those espousing views deemed unacceptable by China. Similarly, Cai and Xu [21] examined 126 texts related to the origin tracing of COVID-19 in the regular press conferences of MFAC. Their research reveals that the spokespersons mainly used a discussive strategy of “seeking similarity”, particularly by adeptly citing authoritative figures, such as scientists and World Health Organization, to win international audiences to endorse China’s stance. To examine how the spokespersons manage China’s image, Wu and Feng [22] analyzed the discursive strategies in tweets related to COVID-19 by two MFAC spokespersons. Their findings suggest that the spokespersons used offensive strategies to counter accusations from the US while concurrently cultivating a positive national image through communicative strategies.

However, previous research on the discourse of MFAC spokespersons has primarily focused on identifying features of discursive strategies, while paying relatively little attention to the underlying factors shaping these strategies, particularly in the context of COVID-19 and the context where the MFAC spokespersons were described as “wolf warrior diplomats” by Western politicians and media [23–25]. Although some few studies have discussed the influence of spokespersons’ discourse on constructing China’s national image, they often fail to provide adequate empirical evidence, typically presenting limited or fragmented examples. It is thus essential to employ a more extensive dataset to substantiate these earlier findings. Moreover, research is also needed to investigate how specific social realities are contested and constructed internationally through spokespersons’ discourse, particularly highlighting contrasting portrayals of national images between China (i.e., Self) and other countries (i.e., Others), especially Western, thereby shedding light on China’s struggle for discursive power in the international arena.

To address the research gap, this paper aims to investigate the features of discursive strategies used by MFAC spokespersons in their discourse on COVID-19 and how these discursive strategies contribute to the diverse representations of national image for both China and other countries in this discursive context. Specifically, this study aims to address the following questions: (1) What discursive strategies do the spokespersons adopt to represent China and other countries in the regular press conferences of MFAC during the COVID-19 pandemic? (2) What features characterize the spokespersons’ construction of national images for China and for other countries in this discursive context? (3) What are the factors, institutional or social, that underpin the different constructions if any?

## Analytical framework: van Dijk’s ideological square

A central concern in critical discourse studies is the relationship between discourse and ideology. As van Dijk [6,26] argues, discourse constitutes one of the most important forms of social practice through which ideologies are shaped, maintained, and transformed, and “if we want to know what ideologies actually look like, how they work, and how they are created, changed and reproduced, we need to look closely at their discursive manifestations” (original emphasis). This perspective foregrounds discourse not merely as a channel of communication but as the primary site where ideologies are produced, circulated, and contested. It also emphasizes that ideological meaning can only be understood in relation to the broader socio-political context in which texts are produced, interpreted, and recontextualized.

Against this theoretical backdrop, a number of analytical frameworks have been developed in discourse analysis that offer complementary tools for examining diplomatic discourse, many of which are methodologically compatible with a social-cognitive understanding of ideology. Proximization Theory [27], for instance, elucidates how political actors discursively construe external actors or events as spatially, temporally, or axiologically imminent in order to legitimize policy positions, a strategy also observed in contemporary Chinese diplomatic discourse [28]. Within this broader landscape, van Dijk’s [26, 267; 29] Ideological Square model occupies a pivotal position. This theoretical framework foregrounds the role of shared mental models and social representations in mediating between discourse structures and ideological systems, thereby offering a principled explanation of how ideological meanings are cognitively stored, reproduced, and strategically activated in diplomatic communication -- most notably through recurrent discursive “us versus them” constructions.

In this study, ideology is understood not as a fixed set of opinions, but as the foundational beliefs underlying the shared social representations of specific kinds of social groups that are in turn the basis of discourse and other social practices [29]. The Ideological Square model posits that ideological discourse is structured around systematic group polarization and realized through four strategies: (1) Express/emphasize information that is positive about Us; (2) Express/emphasize information that is negative about Them; (3) Suppress/de-emphasize information that is positive about Them; (4) Suppress/de-emphasize information that is negative about Us. The Ideological Square model has two core features: firstly, it emphasizes that ideology is group-based. As van Dijk [26, 129] argued, “a group self-schema is the core of all ideologies” and ideologies are not individual, idealistic constructs, but the social constructs that are shared by a group. Secondly, ideology is self-serving, meaning that “ideology-driven social practices typically serve self-interests to the maximum extent” [26, 68–69].

The discursive representation of national images in the MFAC spokespersons’ remarks undoubtedly reflect China’s distinctive diplomatic philosophy and its ideologies. It is thus interesting to examine how the MFAC spokespersons’ pandemic discourse and the discursive strategies they used reflect China’s diplomatic philosophy and the interplay between representation of national images and the discursive structure of China’s diplomatic ideology.

Here national image is understood as a discursively constructed phenomenon that emerges from recurrent patterns of evaluation in institutional discourse. Rather than taking national image as a reflection of sentiment, we operationalize it through linguistic indicators, including collocational tendencies, evaluative orientation, and patterns of attribution associated with specific national actors.

## Methodology

The study of discourse is indispensable for analyzing how ideologies are constructed, maintained, and transformed. By tracing the linguistic and discursive mechanisms through which ideas are framed and represented, we can reveal the subtle ways in which political interests are legitimized. The methodological implication of discourse analysis is that uncovering ideology requires systematic attention to the regularities and patterned structures in discourse. However, discourse analysis *per se* has been criticized on the grounds that discourse analysis cannot eliminate the personal bias of analysts and “the analysis is expediently used in support of interpretation” [30, 128].

To respond to such criticism, Chilton *et al*. [31] propose either explicitly acknowledging the analyst’s normative position on the grounds that no research is objective and all scientific research is subservient to interests; or basing studies on a large corpus of data, rather than on a handful of texts, to support their claims.. One of the advantages of corpus-based approach to discourse analysis, as Baker [10,32] argues, is that it can help identify the repeated patterns conveying meanings that are shared by a discourse community rather than just personal and idiosyncratic and thus help reduce research bias. Baker *et al*. [33] illustrate that combining critical discourse analysis with corpus linguistics makes it possible to reveal hidden ideological patterns by examining the statistical salience and co-occurrence of key lexical items across large datasets. These approaches converge on the view that ideology is not abstract, but materially realized in discourse through recurring lexical, syntactic, and rhetorical structures.

### Data collection and corpus compilation

A corpus-based method was adopted to achieve the above objective. The corpus built for this research comprises transcripts of the MFAC’s regular press briefings during the first two years of the COVID-19 pandemic, spanning from January 2020 to December 2021. These transcripts are available at the official website of the Ministry of Foreign Affairs of China (https://www.fmprc.gov.cn/eng/xw/fyrbt/). Given the aims of this research, the corpus includes only responses from MFAC spokespersons and excludes all journalists’ questions, as the primary interest lies specifically in the spokespersons’ discourse rather than question-and-answer interaction strategies.

To facilitate efficient data retrieval, all Chinese responses were segmented using SegmentAnt_jieba. Such segmentation is required because Chinese, unlike alphabetic languages such as English, is not separated by spaces between words, which otherwise makes it incompatible with corpus analysis software such as WordSmith. Ultimately, the resulting corpus containing MFAC spokespersons’ responses reached a size of 782,593 words. A widely used corpus package software WordSmith (8.0) was chosen to identify collocates. Here the default setting of WordSmith (8.0) was adopted for the generation of collocational lexical items, i.e., minimum frequency at two within a window span from left five to right five stopping at sentence break. For the purpose of calculating collocational relationship, WordSmith (8.0) statistically compares the target corpus against a reference corpus, applying statistical measures like MI^3^. Here *Torch2019*, a balanced corpus consisting of one million Chinese words, compiled from a period closer to the timeframe of the diplomatic discourse under investigation served as the reference corpus.

This particular dataset was selected for two reasons. Firstly, COVID-19 had an extensive global impact, with international discussions and debates about the pandemic becoming prominent since its outbreak. Secondly, in the context of unprecedented global transformations, certain studies have observed a growing assertiveness in MFAC spokespersons’ discourse, leading Western politicians and media to label them as “wolf warrior diplomats” [23–25]. It is therefore valuable to investigate the actual features of this specific type of diplomatic discourse as produced by MFAC spokespersons during press briefings.

However, it is noted that the MFAC spokespersons’ discourse constitutes a highly institutionalized genre, which is shaped by institutional goals, diplomatic conventions, role constraints, and strategic considerations. So the discourse analyzed here does not represent individual attitudes but rather institutional positions. It is also noted that the analysis reports raw frequencies and distributional patterns to describe discursive tendencies within the corpus. As the dataset constitutes a complete collection of institutional texts within a defined period rather than a probabilistic sample, the study prioritizes descriptive statistics over inferential testing.

## Results

A wordlist of the corpus was generated using WordSmith (8.0) and then compared with a list of all the 233 countries/regions in the world, resulting in the identification of 175 countries/regions in the corpus. The five permanent members of the United Nations Security Council were selected for focused analysis because of their central role in global governance. Rather than aiming for universal generalization across all countries, this study adopts an analytically focused case-selection strategy to examine how ideological polarization and national image representations operate in Chinese diplomatic discourse, specifically the MFAC spokespersons’ responses. It is to note that, in addition to official country names, Chinese diplomatic discourse also employs alternative terms to refer to countries. For instance, the spokespersons frequently used “美方” (the US side) to denote the US. Consequently, when utilizing WordSmith for data retrieval, both “美国” (the US) and “美方” (the US side) were employed as the search terms to extract concordance lines of the US. This procedure is also applied to the other four countries. The frequencies for respective countries in the corpus are presented in Table 1.

**Table 1:** Frequencies of the five countries in the MFAC spokespersons’ responses.

	China	The US	Russia	The UK	France
Frequency	20,352	8,196	318	482	163

To capture features of the discursive representation of national images, we conducted further analysis of collocations of the search items. If the collocate of a certain country exhibits positive connotation, this indicates that the country is positively represented in a concordance line. On the contrary, if the collocate manifests negative connotation, this suggests that the country’s image was represented in a negative way.

For example, in the concordance line “We noticed that yesterday **Russia** successfully held a grand military parade to commemorate the 76^th^ anniversary of the victory in the Great Patriotic War”, the collocate “successfully” of “Russia” indicates a positive semantic orientation, representing Russia positively in the concordance line. On the contrary, in the concordance line “The actions of **the US** seriously violate international law and the basic norms of international relations, severely interfere with Sino-US relations and bilateral normal exchanges”, the collocate “violate” associated with “the US” sends out a negative connotation, thus depicting a negative image of the US in this context. With *Torch2019* as the reference corpus, the lists of collocates for the five countries were generated respectively using WordSmith within the span of 5 words to the left and 5 to the right of the search items as shown in Table 2. The collocates are sorted in descending order based on the MI^3^ statistical results.

**Table 2:** Collocational features of the five countries.

	China	The US	Russia	The UK	France
Number of the collocates	1878	903	63	77	46
Frequency of the collocates	14035	4752	360	276	147

An initial examination of the collocates for each country revealed that many of them were neutral and objective expressions without explicit emotional connotations. To obtain collocates that reflect emotional polarity, two additional steps were taken. Firstly, two native Chinese speakers independently coded all the collocates to identify positive and negative ones. It turns out that the results coded by the two native Chinese speakers were generally consistent. For example, in the case of collocates of “China”, coder A picked out 385 positive collocates, while coder B identified 389 positive ones. There were 341 shared positive collocates in their coding, accounting for 89% of coder A’s result and 88% of coder B’s. Similarly, coder A confirmed 176 negative collocates, while coder B marked 185 as negative collocates. There were 163 shared negative collocates between them, accounting for 93% of coder A’s result and 88% of coder B’s. These results indicate that using the shared collocates of different polarities by both coders can accurately reflect the overall situation.

It is noted that the coding of evaluative orientation as positive or negative reflects relative evaluative positioning within diplomatic discourse. Positive orientation refers to discursive patterns that foreground legitimacy, responsibility, cooperation, or alignment with shared norms, whereas negative orientation refers to patterns that emphasize norm violation, irresponsibility, or delegitimization. Importantly, conventional diplomatic expressions were not automatically coded as negative; their evaluative status was determined through contextual and pragmatic analysis of concordance lines. Table 3 presents detailed information of the collocates of the five countries.

**Table 3:** Polarities of each country’s collocates agreed by both coders.

	China		The US		Russia		The UK		France	
	Pos	Neg	Pos	Neg	Pos	Neg	Pos	Neg	Pos	Neg
Coder A	385	176	93	191	6	2	6	9	0	1
Coder B	389	185	77	182	7	3	5	8	0	1
**Both Agreed**	**341**	**163**	**73**	**173**	**6**	**2**	**5**	**8**	**0**	**1**
Proportion for Coder A	89%	93%	78%	91%	100%	100%	83%	89%	/	100%
Proportion for Coder B	88%	88%	95%	95%	86%	67%	100%	100%	/	100%

Secondly, due to syntactical differences between Chinese and English, there are a few exceptional cases that should be excluded in collocate identification. Taking “中国” (China) as an example, although an item may be identified by WordSmith as a collocate of “中国” based on syntactical properties, it may not necessarily be so semantically. For instance, in the line “我们注意到，美方粗暴干涉中国内政、蛮横无理宣布所谓制裁的行径遭到了包括香港在内的中国人民的一致强烈反对和谴责” (We noticed that the US side’s rude interference in China’s internal affairs and its arrogant and unreasonable announcement of so-called sanctions have been unanimously opposed and condemned by the Chinese people, including those in Hong Kong), while the act “蛮横无理宣布” (arrogant and unreasonable announcement) is automatically identified as a (negative) collocate of “China” based on syntactical features, its actual agent semantically is “the US side” rather than “China”.

Therefore, to accurately capture the agent of an act regarding the collocates and to describe the discursive representation of the five countries’ images, we used the collocates as search items, with each country serving as the contextual term, to extract the concordance lines. We then conducted close reading to manually determine the actual agents through the co-text of each concordance line. For example, though “蛮横无理宣布” is initially identified as a negative collocate of “China”, the actual agent of the act is “the US side”. Therefore, “蛮横无理” is not included in the discursive representation of China’s image, but in that of the US. After this filtering procedure, the results were achieved as presented in Table 4.

**Table 4:** Image representation of the five countries.

	China	The US	Russia	The UK	France
Frequency	20352	8196	318	482	163
Pos	3310	12	10	0	0
Neg	0	1400	0	38	0

According to Table 4, apart from China, the US is the second most frequently mentioned country in the spokespersons’ responses, nearly nine times the sum of the other three countries combined. This indicates that the US received high attention in the regular press conferences of MFAC. The result also suggests the US’ significant position in the world order, and the relationship between China and the US being a focal point of public attention domestically and internationally. Meanwhile, it is evident that the positive image of China represented in the spokespersons’ responses stands in sharp contrast to the negative ones for the US and the UK. In particular, the US is negatively represented as much as 1,400 times, accounting for 97% of all the negative representations of Other countries, although there are also a few instances of positive representations of the US. The result thus indicates a consistent pattern in the spokespersons’ discourse that fulfills an ideological function of positive self-presentation and negative other-presentation.

However, it is also obvious that in the spokespersons’ responses, France, albeit a Western country, was portrayed in a neutral way, with neither positive nor negative representations. Meanwhile, Russia, though also seemingly an “Other”, was discursively represented with a straightforwardly positive orientation.

Overall, the corpus evidence thus confirms that national image construction in the MFAC spokespersons’ discourse follows systematic evaluative patterns consistent with ideological polarization as theorized in van Dijk’s Ideological Square. However, the observed variations across different countries also indicate that China dynamically modulates its ideological positioning in response to contextual factors.

## Discussion

As an integral part of the social practice of China’s diplomacy, the spokespersons’ discourse reflects a highly patterned construction of national images. As demonstrated in the Results section, the representations of China and other major countries are characterized by clear asymmetries in frequency and evaluative polarity, suggesting a positive presentation of China, i.e., Self, and a negative presentation of other countries, i.e., Other. Such a result shows that the diplomatic discourse of the spokespersons is ideologically shaped, foregrounding Self in a positive manner and Others negatively. It also suggests that such discourse serves Self’s/China’s interests, fulfilling the egoistic nature of ideology.

However, it is notable that the negative representation of other countries predominantly targets the US and the UK, yet a positive presentation of Russia is observed. This further validates Li and Hu’s [34, 177] argument that some so-called Others are speculative extension of the Self. It also indicates that the identities of Self and Other in the Ideological Square model are not fixed but subject to dynamic changes driven by the self-serving principle.

These regularities provide empirical support for interpreting the spokespersons’ discourse through van Dijk’s Ideological Square, while also pointing to dynamic modulations shaped by institutionalized diplomatic ideology, dynamic geopolitical relations, and culturally embedded communicative norms.

### Diplomatic ideology and the MFAC spokespersons’ discourse

The most salient discursive pattern emerging from the corpus is the overwhelmingly positive representation of China, i.e., Self. As shown by the collocational analysis, China is associated with many positively evaluated collocates, while no negative evaluations remain after agent-based filtering. This extreme skewness is not accidental but reflects a systematic discursive orientation toward positive self-presentation. In contrast, the United States exhibits the highest number of negative collocates, accounting for the vast majority of all negative representations of other countries in the corpus.

Rather than reading this pattern as a mere reflection of political confrontation, it is more analytically productive to interpret it as the discursive realization of China’s diplomatic ideology under conditions of heightened international contestation. In this discursive context, China and the US-led Western countries hold two distinct diplomatic ideologies. China adheres to the diplomatic principles that center on peace, development, cooperation, and mutual benefit, and advocates for the establishment of a new mode of international relations based on mutual respect, fairness, justice, and win-win cooperation. This is most obviously manifested in its promotion of the diplomatic concept of building “a community with a shared future for mankind” [35–37]. In contrast, some Western countries, particularly the US, have often articulated critiques of other countries, including China, through discourses of democracy, human rights, and security concerns. For example, the 2017 National Security Strategy described China and Russia as “revisionist powers”. These contextual factors provide a background for interpreting why the US was frequently represented as a salient Other in the MFAC spokespersons’ discourse.

The ideological differences between China and Western countries have, to some extent, resulted in their different measures in addressing the global COVID-19 pandemic. Throughout this period, China was subjected to heightened international scrutiny. A body of scholarship on Western media discourse suggests that some news outlets and political-media ecosystems frequently framed China through lenses of blame attribution, including the circulation of racially marked labels such as “Chinese virus” and “Wuhan virus” and related stigmatizing narratives [38–39]. Research on European popular press further indicates recurring patterns of blame allocation in which responsibility for the outbreak and its consequences was discursively attributed to China [40]. Comparative and semiotic analyses further show how pandemic reporting could become politicized, with China’s domestic mitigation measures and international assistance sometimes interpreted through strategic or ideological frames rather than primarily public-health considerations [41]. In this light, such media framings may be understood as aligning with broader Western political efforts to exert pressure on China and to constrain its national rejuvenation agenda [42–43].

Danziger and Schreiber [44] suggest that, much like interpersonal interactions, a country’s international communication entails using discourse to project its values and norms, thereby managing its global image and securing international support. To mitigate negative perceptions globally during the COVID-19 pandemic, it is crucial for China to actively amplify its voice internationally and cultivate a favorable image as a significant global actor, with diplomatic discourse serving as a key instrument in this process. The spokespersons’ discourse consistently frames China as responsible, cooperative, and constructive, while the US and, to a lesser extent, the UK-based BBC are recurrently associated with negatively evaluated predicates such as *violate*, *interfere*, *smear*, and *irresponsible*. Such contrastive evaluation aligns closely with the ideological function identified by van Dijk [26, 267], whereby discourse serves group interests by presenting positive image of Self and negative image of others.

On the other hand, the occasional cases of positive evaluation of the US, though quantitatively marginal, indicate that ideological polarization is not absolute. This selective patterning supports the interpretation that MFAC spokespersons’ discourse operates within an institutional logic that balances ideological positioning with diplomatic pragmatism. In this sense, diplomatic ideology is not merely asserted but is continuously negotiated through discourse in response to international criticisms.

### Geopolitical relations and diverse representations of national image

The differentiated representations of the five permanent members of the UN Security Council can be further explained by reference to geopolitical relations, as evidenced by the distributional patterns in the corpus. The high frequency of references to the US and the UK-based BBC, together with the predominance of negatively connotated collocates associated with them, indicate that they are the principal discursive Others in this dataset and the primary discursive axis around which negative national image construction is oriented.

As the two largest global economies, China and the US have a significant foundation for cooperation, yet they also experience geopolitical tensions and conflicts of interest.US official strategic documents during the Trump administration increasingly framed China as a major strategic challenge. For example, the *2018 National Defense Strategy* (https://media.defense.gov/2020/May/18/2002302061/-1/-1/1/2018-NATIONAL-DEFENSE-STRATEGY-SUMMARY.PDF) explicitly stated that “China is a strategic competitor”, declaring that the competition between the US and China is strategic, comprehensive, and long-term.

Amid growing tensions in Sino-US relations, Chinese official discourse has repeatedly stressed the importance of being prepared for struggle. Poh and Li [45] observed that both the academic and political fields have increasingly acknowledged the strengthening of China’s external messaging and discursive capacity regarding its diplomatic and security interests in recent years. This viewpoint was echoed by the remarks of Mao Ning, an MFAC spokesperson, during the Third China Spokesperson Forum (May 27, 2023), where Mao highlighted that in the face of relentless defamation campaigns by anti-China hostile forces, the spokespersons refused to be silent lambs or gentlemen but worked as warriors to proactively defend China’s interests, image, and dignity in the fiercely contested international discursive arena.

**Fig 1:**
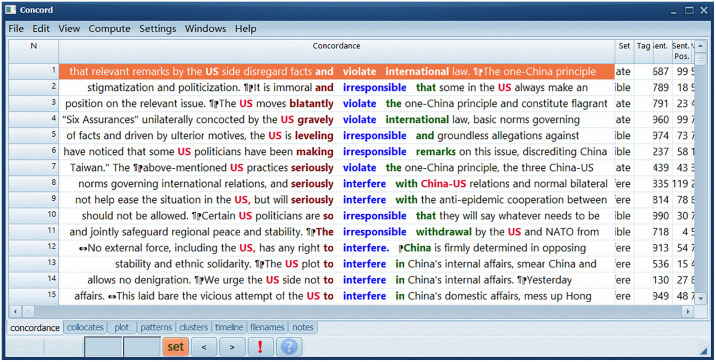
Concordance lines of “violate”, “interfere” and “irresponsible” with “US”.

Inspired and guided by this ideological orientation, the spokespersons had engaged in a tit-for-tat struggle in the frontline of diplomacy. As part of these diplomatic practices, the spokespersons used offensive discursive strategies to respond to criticisms from the US government and certain Western media outlets, which were represented in the spokespersons’ discourse as attempts to stigmatize or discredit China’s pandemic response. Through recurrent collocates and concordance lines involving terms such as “violate”, “interfere”, and “irresponsible” as can be seen in Fig 1, the spokespersons’ discourse represented the US in negative evaluative terms, particularly in relation to pandemic governance, international responsibility, and intervention in other countries’ affairs. By contrast, China was frequently associated with positively evaluated terms related to cooperation, responsibility, mutual assistance, and multilateralism. These contrastive patterns are consistent with the quantitative distribution reported in Table 4, where China received 3,310 positive representations, whereas the US received 1,400 negative representations. Through these discursive efforts, the spokespersons actively engage in China’s diplomatic practices with the ideological purpose of upholding China’s national interests and image as well as strengthening China’s legitimacy in geopolitical competition.

In contrast to the US and the UK, Russia is represented in a markedly different manner. Although it is formally categorized as an Other within the Ideological Square framework, the corpus reveals a consistent tendency toward positive evaluation, albeit at a much lower frequency overall. The relatively positive representation of Russia may be interpreted in relation to the broader context of China-Russia strategic cooperation. In fact, China-Russia relations have in many years involved sustained strategic coordination and that Russia occupies a high-ranking position in China’s partnership diplomacy. Notably, both countries face similar external pressures and challenges, leading to a shared commitment to safeguarding their sovereignty and interests. It is therefore not difficult to understand the positive representation of Russia in the spokespersons’ responses, i.e., Russia is largely represented as a Self rather than an Other. This pattern supports the view that the boundaries between Self and Other are not fixed categories but are constantly (re)configured in accordance with geopolitical alignment. Within van Dijk’s framework, Russia thus emerges as a partial Self or ideologically aligned actor, which explains its favorable portrayal despite its formal status as an external Other.

The findings suggest that the discursive construction of national images is closely shaped by geopolitical relations, which provide an important contextual backdrop for how different countries are presented in Chinese diplomatic discourse. These discursive representations, in turn, may contribute to framing mutual perceptions and expectations, thereby influencing the broader dynamics of China’s relations with other countries.

### Traditional Chinese culture and contemporary China’s diplomacy

Beyond ideological polarization and geopolitical relations, the diverse representations of national images through the MFAC spokespersons’ responses are deeply embedded in traditional Chinese culture. Confucianism, in particular, has significantly influenced China’s political, social, and ethical norms, laying an essential foundation for contemporary Chinese diplomatic discourse [46–47].

Central Confucian tenets such as harmony (和), benevolence (仁), righteousness (义), and propriety (礼) inform China’s approach to international relations, emphasizing harmonious coexistence, mutual respect, and peaceful development. These traditional philosophical thoughts offer China a unique conceptual toolkit to manage complex international relations and guide its diplomatic engagements [48,49].‌‌ Selected elements of traditional Chinese cultural thoughts, such as harmony, righteousness, and propriety, may provide a broader interpretive lens for understanding the coexistence of cooperative and confrontational elements in MFAC spokespersons’ discourse. For example, the Confucian principle of harmony may help contextualize China’s emphasis on peaceful diplomacy, dialogue, and cooperation. At the same time, notions of righteousness and propriety may help explain how criticism of perceived injustice, unilateralism, or hegemonic behavior is morally framed within diplomatic discourse.

On the one hand, the positive representation of China is predominantly constructed through lexemes associated with cooperation, shared responsibility, and collective action, as evidenced by the high frequency and consistency of such collocates in the China-related concordance lines. This recurrent preference for relational and cooperative framing can be interpreted as resonating with the Confucian emphasis on harmony, which privileges cooperation over direct confrontation. The stability of this pattern across the corpus suggests sustained discursive orientation rather than a response to isolated situational demands. Within this discursive logic, China is routinely framed as favoring dialogue in conflict management, pursuing mutually beneficial outcomes through negotiation, and promoting long-term relational frameworks such as the diplomatic notion of “a community with a shared future for mankind”. Within this discursive philosophy, China is framed as favoring resolution through dialogue rather than conflict, pursuing mutually advantageous outcomes via peaceful negotiations, and advocating the building of new and reciprocal international relationships.

On the other hand, the selective and context-dependent use of offensive discursive strategies further supports a culturally informed interpretation of the spokespersons’ discourse. Corpus evidence shows that such strategies are overwhelmingly concentrated in references to specific actors -- most notably the United States and the UK-based BBC. The concentration of negative evaluative suggests that offensive discursive strategies were not evenly distributed across all external actors. Rather, they were associated primarily with actors as involved in criticism, blame attribution, or perceived challenges to China. It indicates that the spokesperson’ adoption of the Confucian notion of righteousness, whereby injustice or hegemonic behavior in global politics should be opposed. This is reflected in China’s unwavering efforts to safeguard its national interests and its commitment to vigorously responding to any diplomatic attempts to discredit or suppress it. Specifically, the MFAC spokespersons adopted various offensive discursive strategies to counteract misunderstandings and negative representation of China stemming from Western countries’ zero-sum mentality. By contrast, when addressing countries positioned as cooperative, such as Russia, the spokespersons employ positively oriented diplomatic rhetoric, underscoring the conditional and relational nature of evaluative positioning in the corpus.

## Conclusion and future research

Drawing on a corpus-based discourse analysis approach, this paper investigates the discursive construction of images of the five permanent members of the United Nations Security Council in the MFAC spokespersons’ responses during the regular press conferences. The research results indicate that the spokespersons used a combination of communicative and offensive discursive strategies in their diplomatic discourse. On the one hand, the spokespersons’ discourse constructed a positive image of China through communicative strategies, recurrently associating China with cooperation in the global pandemic response. On the other hand, the spokespersons’ discourse also included offensive strategies in response to negative narratives, accusations, and criticisms directed at China primarily by the US and some other western media such as BBC. These offensive strategies involved openly criticizing the ineffective pandemic governance of the US-led western countries and condemning what the MFAC spokesperson, Zhao Lijian, described as hegemonic conducts and unwarranted interference in the internal matters of sovereign nations during the Regular Press Conference on July 12, 2021.

In the spokespersons’ responses, China as Self was positively presented while the other countries were portrayed generally in a negative manner, aligning with van Dijk’s Ideological Square model. However, the negative presentation of Others primarily targeted the US and the UK. As a contrast, while seemingly being depicted as an Other, Russia was discursively presented in a positive way. The divergence in discursive presentation of Self and Others by the MFAC spokespersons, are closely tied to ideological factors underpinning China’s diplomacy, which are a result of the joint influence of current geopolitical dynamics and Confucianism. Beyond the specific geopolitical context of the COVID-19 pandemic, the findings of this study contribute to broader discussions on how ideology is discursively operationalized in institutional diplomatic communication. By identifying systematic patterns of evaluative asymmetry and relational positioning across a corpus of spokesperson discourse, the analysis demonstrates how ideological polarization is not merely asserted but routinized through recurring linguistic choices. In this sense, the study shifts the focus from reactive responses to particular actors to the underlying discursive mechanisms through which national images and ideological positions are dynamically constructed and negotiated.

This research adds to ongoing debates in corpus-based discourse analysis and diplomatic communication, particularly regarding the discursive construction of national images in the Chinese diplomatic context. It illustrates the ways in which the integration of van Dijk’s Ideological Square with corpus-based collocational and evaluative analysis strengthens the empirical operationalization of ideological discourse in Chinese diplomatic contexts. Beyond its theoretical contributions, the findings also carry some practical implications for diplomatic communication and national image management. The analysis shows that cooperative and offensive discursive strategies used to represent national images in spokesperson discourse were unevenly distributed across the national actors and these observed distributional differences may be interpreted in relation to broader geopolitical dynamics. From a policy perspective, this suggests that effective diplomatic communication requires balancing assertiveness and relational framing. Emphasizing cooperation, responsibility, and shared interests, while juxtaposing the Self with the majority, may help enhance international credibility and manage national image risks during global crises.

However, it should be noted that the present analysis focuses on a specific group of countries with exceptional geopolitical significance. As such, the findings are not intended to be generalized to all countries mentioned in the spokespersons’ discourse. These discursive patterns may be applicable to other settings, although their concrete manifestations are likely to vary depending on geopolitical relations.

Future research may benefit from incorporating alternative analytical frameworks, such as appraisal theory or narrative approaches, to complement the corpus-based and ideological analysis in this study. Further studies could also extend the research scope and methodologically include coder reliability metrics such as Cohen’s Kappa and inferential statistics such as chi-square test for comparative studies. For instance, a comparative analysis of diplomatic discourses by China and the United States would likely yield valuable insights into their respective discursive strategies. An exploration into how the national images of G20 countries are depicted within Chinese diplomatic narratives, including examining when and why these representations are framed positively or negatively, would offer a deeper understanding of the intricate relationship between ideological frameworks and diplomatic practices.
