# Supplementary material for: Representing national images of self and others through China’s diplomatic discourse: A corpus-based study
Source: PLoS One. 2026 Jun 12;21(6):e0350726. doi: 10.1371/journal.pone.0350726 (PMC13262881; doi:10.1371/journal.pone.0350726)
Supplement: Attachment — Submitted filename: 5.6_Response to Reviewers.docx [file pone.0350726.s005.docx]

Journal Office requests: During internal review of this submission, PLOS ONE staff editors identified several sentences in the manuscript that may affect its ability to meet the journal’s publication criteria (https://journals.plos.org/plosone/s/criteria-for-publication). To ensure the manuscript maintains a neutral and objective tone, that all statements are appropriately supported by peer-reviewed literature or the presented results, and that claims are not overstated, we recommend revising the text accordingly.

We thank the editor(s) for drawing our attention to this important issue. We’ve carefully reviewed the manuscript throughout, with particular attention to the Discussion, and Conclusion sections, and revised the relevant passages accordingly.

Specifically, it is not clear from the presented results how the following statements are supported by the work. We ask authors to revise the text or provide an appropriate supporting citation.

1. "In contrast, Western countries, particularly the US, seek unilateralism in the name of democracy, freedom, and human rights. They engage in interventions in the internal affairs of other countries and scramble to maintain the hegemonic position and vested interests of developed Western countries in global governance."

Thanks. We’ve removed “They engage in interventions in the internal affairs of other countries and scramble to maintain the hegemonic position and vested interests of developed Western countries in global governance.” We’ve also reworded them as you can see in the manuscript.

“In contrast, some Western countries, particularly the US, have often articulated critiques of other countries, including China, through discourses of democracy, human rights, and security concerns. For example, the 2017 National Security Strategy described China and Russia as “revisionist powers”. These contextual factors provide a background for interpreting why the US was frequently represented as a salient Other in the MFAC spokespersons’ discourse.”

2. "Politicians, including president of the US, initiated trade wars and suppressed Chinese enterprises and depicted China as a threat and competitor in their public speeches. In official documents including The National Security Strategy Report, The National Defense Strategy Report, and The Nuclear Posture Review, the Trump administration explicitly labeled China as a “strategic competitor”, declaring that the competition between the US and China is strategic, comprehensive, and long-term"
2a. We also request that links are provided to the above named reports

Thanks. We’ve revised this passage to avoid unsupported or politically evaluative wording. We now refer more narrowly to official US strategic documents and provide links to the relevant reports, as requested.

“US official strategic documents during the Trump administration increasingly framed China as a major strategic challenge. For example, the 2018 National Defense Strategy explicitly stated that “China is a strategic competitor”, declaring that the competition between the US and China is strategic, comprehensive, and long-term.

3. "As part of diplomatic practices, they adopted offensive strategy to criticize the US government and politicians who have wantonly smeared China’s pandemic prevention efforts and projected a negative image of the US."

Thanks. We’ve revised the sentence to distinguish between the authors’ analysis and the spokespersons’ own discursive representation. The revised wording now states that the spokespersons represented certain US criticisms as attempts to stigmatize or discredit China, rather than presenting this as the authors’ own judgment.

“As part of these diplomatic practices, the spokespersons used offensive discursive strategies to respond to criticisms from the US government and certain Western media outlets, which were represented in the spokespersons’ discourse as attempts to stigmatize or discredit China’s pandemic response.”

4. "Through descriptions and evaluations of its unilateralist and imperious behaviors, the spokespersons cast the US in a negative light who is irresponsible domestically for the ineffective pandemic prevention and hegemonic internationally with extended jurisdiction to interfere in other countries’ internal affairs. By contrast, the spokespersons brought to the fore China’s diplomatic principles of multilateralism and win-win cooperation, thereby constructing a favorable image of China as a responsible and cooperative country and highlighting China’s divergence with the West in ideology."
4a. It is unclear that sufficient evidence has been provided in the results themselves to support these statements. As a suggestion, quotes from the spokespersons may provide support.

Thanks. We’ve revised the passage so that terms such as “unilateral”, “irresponsible”, and “interference” are clearly presented as evaluative categories found in or inferred from the spokespersons’ discourse, rather than as the authors’ direct characterization of the US. We have also added representative concordance examples to show how negative evaluations of the US were linguistically realized.

“Through recurrent collocates and concordance lines involving terms such as “violate”, “interfere”, and “irresponsible” as can be seen in Figure 1, the spokespersons’ discourse represented the US in negative evaluative terms, particularly in relation to pandemic governance, international responsibility, and intervention in other countries’ affairs. By contrast, China was frequently associated with positively evaluated terms related to cooperation, responsibility, mutual assistance, and multilateralism. These contrastive patterns are consistent with the quantitative distribution reported in Table 4, where China received 3,310 positive representations, whereas the US received 1,400 negative representations.”

Figure 1 Concordance lines of “violate”, “interfere” and “irresponsible” with “US”

5. "Their partnership is characterized by frequent high-level exchanges, shared positions on global issues, and a commitment to strengthening cooperation. Notably, both countries face similar external pressures and challenges, leading to a shared commitment to safeguarding their sovereignty and interests"

Thanks. We’ve revised this statement to avoid overgeneralization but to present China-Russia cooperation as a contextual factor that may help explain the relatively positive representation of Russia in the corpus, rather than as a direct causal claim.

“The relatively positive representation of Russia may be interpreted in relation to the broader context of China-Russia strategic cooperation. In fact, China-Russia relations have in many years involved sustained strategic coordination and that Russia occupies a high-ranking position in China’s partnership diplomacy.”

6. "For instance, the Confucian principle of harmony underscores China’s advocacy for peaceful diplomacy, dialogue, and cooperation rather than confrontation. Simultaneously, righteousness and propriety serve as moral justifications for China’s diplomatic stance and its criticism of perceived injustice or hegemonic behavior in global politics. Such cultural elements enable the MFAC spokespersons to balance assertiveness with courtesy, employing both communicative and offensive discursive strategies strategically. This culturally informed diplomatic rhetoric thus not only helps manage China’s international image but also effectively counters external challenges."

Thanks. We’ve revised this passage to make clear that Confucian concepts are used only as a possible interpretive lens, not as a direct causal explanation for the spokespersons’ discursive strategies. We have also removed wording suggesting that these cultural elements “effectively counter” external challenges.

“Selected elements of traditional Chinese cultural thoughts, such as harmony, righteousness, and propriety, may provide a broader interpretive lens for understanding the coexistence of cooperative and confrontational elements in MFAC spokespersons’ discourse. For example, the Confucian principle of harmony may help contextualize China’s emphasis on peaceful diplomacy, dialogue, and cooperation. At the same time, notions of righteousness and propriety may help explain how criticism of perceived injustice, unilateralism, or hegemonic behavior is morally framed within diplomatic discourse.”

7. "The systematic recurrence of negative evaluative framing in these cases suggests that offensive strategies are not indiscriminately applied but are activated in response to perceived challenges or accusations directed at China"

Thanks. We’ve revised this sentence to avoid making a strong causal claim about strategic intention.

“The concentration of negative evaluative suggests that offensive discursive strategies were not evenly distributed across all external actors. Rather, they were associated primarily with actors as involved in criticism, blame attribution, or perceived challenges to China.”

Conclusions:

8. "On the one hand, they sought to uphold and enhance China’s positive international image by stressing the scientific rigor underlying virus origin investigations and promoting global cooperation in combating COVID-19 as a shared global challenge."
8a. It is not currently clear that this statement is supported by the results presented in the manuscript. We therefore ask the authors to ensure that it is adequately substantiated, either by the study’s findings (e.g., through the inclusion of relevant quotations) or by appropriate references.

Thank you. We’ve revised the conclusion to align it more directly with the corpus findings reported in this study, particularly the positive collocational patterns associated with China. While the spokespersons’ discourse includes cases emphasizing the scientific rigor of virus origin investigations, we’ve reformulated the conclusion to avoid giving undue prominence to claims not directly foregrounded in the Results section.

“On the one hand, the spokespersons’ discourse constructed a positive image of China through communicative strategies, recurrently associating China with cooperation in the global pandemic response.”

9. "On the other hand, they skillfully adopted"
9a. This phrase appears to include subjective language. Please clarify whether the use of the term “skillfully” is justified within the scope of the study. If not directly supported by the results or appropriate references, we recommend revising this phrasing to ensure a more neutral and objective tone.

Thanks. We’ve removed the word “skillfully” to make it more neutral.

“On the other hand, the spokespersons’ discourse also included offensive strategies in response to negative narratives, accusations, and criticisms directed at China primarily by the US and some other western media such as BBC.”

10. "and defamation attempts spearheaded primarily by the US and some other western media such as BBC."

Thanks. We’ve reworded the sentence. “defamation attempts spearheaded” has been removed as you can see from our response to comment 9.

11. "The analysis shows that national image construction in spokesperson discourse relies on a strategic calibration of cooperative and confrontational strategies, which are selectively deployed in response to geopolitical dynamics and perceived external challenges."
11a. Please provide supportive evidence that the spokesperson discourse was selectively deployed for the stated reasons. If this interpretation is not directly supported by the results or appropriate references, we recommend revising the statement to ensure it accurately reflects the evidence presented.

Thanks. We’ve revised the sentence to avoid making a strong claim that is not very directly foregrounded in the Results section. Since the Conclusion section is intended to synthesize the main findings rather than introduce new evidence, we’ve reformulated the statement in more cautious terms.

“The analysis shows that cooperative and offensive discursive strategies used to represent national images in spokesperson discourse were unevenly distributed across the national actors and these observed distributional differences may be interpreted in relation to broader geopolitical dynamics.”

Overall, we have revised the manuscript to ensure that the discussion remains grounded in the corpus results. Where contextual explanations are retained, we’ve reformulated the relevant arguments in more cautious terms and supported them with official documents where appropriate. We’ve also clarified that the analysis concerns representations in the spokespersons’ discourse rather than the authors’ own judgments.

We are very grateful to the editor(s) for these helpful suggestions, which have greatly improved the overall quality of our manuscript.
