# Supplementary material for: Representing national images of self and others through China’s diplomatic discourse: A corpus-based study
Source: PLoS One. 2026 Jun 12;21(6):e0350726. doi: 10.1371/journal.pone.0350726 (PMC13262881; doi:10.1371/journal.pone.0350726)
Supplement: Attachment — Submitted filename: Peer_Review_PLOS_ONE_China_Diplomatic_Discourse.docx [file pone.0350726.s001.docx]

**Peer Review Report**

The study “Representing national images of Self and Others through China’s Diplomatic Discourse: A Corpus-based Study” is timely and relevant, as it addresses the interplay between discourse, ideology, and national image in Chinese diplomacy. It has a Solid methodological framework that combines corpus linguistics with critical discourse analysis using van Dijk’s Ideological Square as a theoretical foundation. However, many areas need to be addressed.

**Comments:**

1. Abstract is dense, lacks methods, and key contributions.
2. Theoretical framework relies heavily on van Dijk without integrating newer discourse theories.
3. Discussion occasionally leans toward political commentary rather than data-based interpretation.
4. The role of Confucian philosophy needs a stronger textual linkage to corpus evidence.
5. Language requires minor proofreading to correct article use and reduce redundancy.

**Suggestions:**

1. Simplify and clarify the Abstract; include corpus details and specify the study’s unique contribution.
2. Clarify the research gap in the Introduction and define 'Self' and 'Other' earlier.
3. Integrate more recent works in critical discourse studies (e.g., multimodal CDA, framing theory).
4. Add coder reliability metrics such as Cohen’s Kappa to enhance methodological transparency.
5. Include a brief justification for choosing the five UNSC countries and discuss implications for generalizability.
6. Ensure Discussion links empirical results directly to ideological interpretation rather than general political commentary.
7. The manuscript would benefit from clearer structural organization. Currently, several sections blend conceptually, making it difficult to distinguish between major components. The Introduction (pp. 4–6) merges literature review material, which should be moved to a separate section titled 'Literature Review' following the Introduction. Similarly, 'Research Design', 'Method', and 'Data Collection' (pp. 7–10) overlap and should be consolidated under a unified 'Methodology' section with subsections such as 'Research Design and Rationale', 'Data Collection and Corpus Construction', and 'Analytical Procedures'. The Results and Discussion (pp. 10–21) intermingle; interpretive commentary should be reserved for the Discussion to maintain analytical clarity. The Conclusion (pp. 22–23) could be reframed as 'Conclusion and Future Research' to emphasize a forward-looking approach.
8. Conclude with theoretical contributions to corpus-based discourse analysis and future comparative research directions.
9. Add a dedicated Limitations subsection addressing corpus scope and exclusion of journalists’ questions.
10. Finally, the manuscript requires careful proofreading to address several minor but recurring issues of grammar, punctuation, and citation style. For example, on pages 6–7, in-text citations such as “van Dijk [19, 267; 20]; [21, 128]” are inconsistently punctuated and formatted. Similarly, within the same section, numbered items in the Ideological Square model— (1) Express/emphasize information that is positive about Us; … (4) Suppress/de-emphasize information that is negative about Us”—contain inconsistent capitalization and spacing around conjunctions (“Us”, “Them”). On page 8, the citation of external web resources— “These transcripts are available at https://www.fmprc.gov.cn/eng/xw/fyrbt/”—should follow standard referencing conventions, ideally formatted as a reference entry or footnote rather than embedded in the running text.

**Need major revisions.**
