# Supplementary material for: Representing national images of self and others through China’s diplomatic discourse: A corpus-based study
Source: PLoS One. 2026 Jun 12;21(6):e0350726. doi: 10.1371/journal.pone.0350726 (PMC13262881; doi:10.1371/journal.pone.0350726)
Supplement: Attachment — Submitted filename: 1.9 Response to Reviewers.docx [file pone.0350726.s003.docx]

Journal Requirements:

When submitting your revision, we need you to address these additional requirements.

1. Please ensure that your manuscript meets PLOS ONE's style requirements, including those for file naming. The PLOS ONE style templates can be found at

<https://journals.plos.org/plosone/s/file?id=wjVg/PLOSOne_formatting_sample_main_body.pdf> and <https://journals.plos.org/plosone/s/file?id=ba62/PLOSOne_formatting_sample_title_authors_affiliations.pdf>

Thank you. We have revised the formats accordingly.

2. We note that the grant information you provided in the ‘Funding Information’ and ‘Financial Disclosure’ sections do not match.

When you resubmit, please ensure that you provide the correct grant numbers for the awards you received for your study in the ‘Funding Information’ section.

Thank you for pointing it out.

3. Thank you for stating the following financial disclosure:

“This is part of the Research Projects of The National Social Science Fund of China (20&ZD140), Humanities and Social Sciences of the Ministry of Education (23YJA740017), Shanghai Pujiang Talent Program (24PJC045).”

Please state what role the funders took in the study.  If the funders had no role, please state: "The funders had no role in study design, data collection and analysis, decision to publish, or preparation of the manuscript." If this statement is not correct you must amend it as needed.

Please include this amended Role of Funder statement in your cover letter; we will change the online submission form on your behalf.

Thank you. The corresponding author, Tao Li, is the CI of the Major Project of The National Social Science Fund of China (20&ZD140), and the PI for the other two projects, i.e., Humanities and Social Sciences of the Ministry of Education (23YJA740017), Shanghai Pujiang Talent Program (24PJC045).

4. Please note that funding information should not appear in the Acknowledgments section or other areas of your manuscript. We will only publish funding information present in the Funding Statement section of the online submission form. Please remove any funding-related text from the manuscript.

Thank you for your reminder. We’ve removed it.

5. Please note that your Data Availability Statement is currently missing the repository name. If your manuscript is accepted for publication, you will be asked to provide these details on a very short timeline. We therefore suggest that you provide this information now, though we will not hold up the peer review process if you are unable.

Thank you. Just checked. It is still working.

<https://github.com/lt0806/Data/blob/main/MFAC_Data-main.zip>

6. Please amend either the title on the online submission form (via Edit Submission) or the title in the manuscript so that they are identical.

Thank you for your reminder.

7.  We note you have included a table to which you do not refer in the text of your manuscript. Please ensure that you refer to Table 4 in your text; if accepted, production will need this reference to link the reader to the Table.

Thank you very much for pointing it out. We’ve deleted two tables which we found not necessary in the previous versions but forgot to change others. Now they are changed.

8. If the reviewer comments include a recommendation to cite specific previously published works, please review and evaluate these publications to determine whether they are relevant and should be cited. There is no requirement to cite these works unless the editor has indicated otherwise.

Thank you for your reminder.

**Additional Editor Comments:**

All three reviewers find you manuscript interesting but suggested some major revision before further porcessing. Therefore, I decided to provide you with an opportunity to revise and resubmit you manscript according.  Reviewers comments are attached with email.

Thank you very much. We’ve revised our manuscript in accordance with your and the reviewers’ suggestions and comments.

**Review Comments to the Author**

Reviewer #1: Peer Review Report
The study “Representing national images of Self and Others through China’s Diplomatic Discourse: A Corpus-based Study” is timely and relevant, as it addresses the interplay between discourse, ideology, and national image in Chinese diplomacy. It has a Solid methodological framework that combines corpus linguistics with critical discourse analysis using van Dijk’s Ideological Square as a theoretical foundation. However, many areas need to be addressed.
Comments:
1. Abstract is dense, lacks methods, and key contributions.

Thank you. I’ve added methods and contributions.

2. Theoretical framework relies heavily on van Dijk without integrating newer discourse theories.

Thank you. We’ve added reviews of the new frameworks of discourse analysis as you can see on pages 6-8. We’ve also included these new citations in the reference part.

3. Discussion occasionally leans toward political commentary rather than data-based interpretation.

Thank you. We’ve revised some parts of it to make the discussion more related to the data. Details can be seen in the whole discussion section.

4. The role of Confucian philosophy needs a stronger textual linkage to corpus evidence.

Thank you. We’ve revised it to improve the connections between the two.

5. Language requires minor proofreading to correct article use and reduce redundancy.
Suggestions:
1. Simplify and clarify the Abstract; include corpus details and specify the study’s unique contribution.

Thank you. We’ve revised it as you suggested above.

2. Clarify the research gap in the Introduction and define 'Self' and 'Other' earlier.

Thank you. We’ve revised it accordingly.

3. Integrate more recent works in critical discourse studies (e.g., multimodal CDA, framing theory).

Thank you. We’ve added some reviews of the new frameworks of discourse analysis as you can see on pages 6-8. We’ve also included these new citations in the reference part.

4. Add coder reliability metrics such as Cohen’s Kappa to enhance methodological transparency.

Thank you. It is true that it might increase reliability of the results. We didn’t use interrater reliability test because we chose only those two coders shared. Anyway, we’ve included this limitation in the conclusion section.

5. Include a brief justification for choosing the five UNSC countries and discuss implications for generalizability.

Thank you. We’ve added a brief justification for choosing the five countries as you can see on page 11. We acknowledge that focusing on the five countries limits the scope of generalizability, which is also included in the conclusion section.

6. Ensure Discussion links empirical results directly to ideological interpretation rather than general political commentary.

Thank you. We’ve revised some parts of it to make the discussion more related to the data. Details can be seen in the whole discussion section.

7. The manuscript would benefit from clearer structural organization. Currently, several sections blend conceptually, making it difficult to distinguish between major components. The Introduction (pp. 4–6) merges literature review material, which should be moved to a separate section titled 'Literature Review' following the Introduction. Similarly, 'Research Design', 'Method', and 'Data Collection' (pp. 7–10) overlap and should be consolidated under a unified 'Methodology' section with subsections such as 'Research Design and Rationale', 'Data Collection and Corpus Construction', and 'Analytical Procedures'. The Results and Discussion (pp. 10–21) intermingle; interpretive commentary should be reserved for the Discussion to maintain analytical clarity. The Conclusion (pp. 22–23) could be reframed as 'Conclusion and Future Research' to emphasize a forward-looking approach.

Thank you. We agree that clearer structure is important for reader accessibility and we’ve revised the manuscript accordingly.

8. Conclude with theoretical contributions to corpus-based discourse analysis and future comparative research directions.

Thank you. We’ve added it as you can see in the conclusion.

9. Add a dedicated Limitations subsection addressing corpus scope and exclusion of journalists’ questions.

Thank you. We’ve added limitations in the conclusion.

10. Finally, the manuscript requires careful proofreading to address several minor but recurring issues of grammar, punctuation, and citation style. For example, on pages 6–7, in-text citations such as “van Dijk [19, 267; 20]; [21, 128]” are inconsistently punctuated and formatted. Similarly, within the same section, numbered items in the Ideological Square model— (1) Express/emphasize information that is positive about Us; … (4) Suppress/de-emphasize information that is negative about Us”—contain inconsistent capitalization and spacing around conjunctions (“Us”, “Them”). On page 8, the citation of external web resources— “These transcripts are available at https://www.fmprc.gov.cn/eng/xw/fyrbt/”—should follow standard referencing conventions, ideally formatted as a reference entry or footnote rather than embedded in the running text.

Thank you. We’ve read through the whole manuscript and revised the formats accordingly. In “van Dijk [19, 267; 20]”, “19” and “20” refer to different citations with “;” to separate them and “267” is page number. 19 is a monograph while 20 is a journal paper which doesn’t require a page number. However, there is one exception about the external web resource without an author. It seems there is no such rule for this kind of reference in PlosOne’s requirement and we don’t know how to deal with it.

Overall, we are very grateful for your detailed and constructive suggestions, which have largely improved the clarity and quality of our manuscript.

Reviewer #2: This is an interesting paper on representing national images of self and others through China’s Diplomatic discourse. The study is based on discursive analysis of spokemen for China’s Ministry of Foreign Affairs during regular press conferences and shows how these discursive strategies shape the national images of China and the other four permanent members of the United Nations Security Council. The results are good and highlight China’s stance and international cooperation initiatives while also using offensive discourse strategy to counter criticisms from US-led Western nations and media regarding the pandemic period as well as some positive self-presentation and negative other-presentation to further justify the rationality of China’s stance and promote China’s image.

Thank you for your positive comments.

Besides its merits, some revisions are needed throughout the paper.
First, the introduction and the conceptual background of the paper should be more grounded in international diplomatic studies and this is also reflected in the scarce reference sources of this paper (32 references are few for a good academic paper!).

Thank you. Following your suggestion, we’ve added a background to strengthen its engagement with relevant scholarship in international diplomatic studies and crisis communication. We also included in the discussion part more engagement with international diplomatic studies. As a result, the reference list has been enriched as well.

Second, the method is anchored in discourse analysis and authors present the advantages of this method but readers need to know more about the limits of the method and data and what were the challenges in collecting those data. More details on how data was interpreted need to be addressed.

Thank you for this important methodological comment. We’ve revised the Methodology section accordingly. We’ve included a paragraph in Methodology and another one in Conclusion section as well, discussing the issues related to institutional genre constraints, the scope of generalizability, and how corpus findings were interpreted by combining quantitative patterns with close qualitative reading.

Third, the discussions related to the results of the paper should be more related to international diplomatic studies.

Thank you. We’ve rewritten the Discussion section and included more discussions in the scope of international diplomatic studies with revisions made particularly on pages from 18 to 24.

Finally, the conclusions are too short. They should also include not only the theoretical perspectives but also the practical dimensions of the results, so some policy recommendations should be mentioned. Also, the broader limitation of the study has to be mentioned in the conclusions.

Thank you for this valuable suggestion. We’ve revised and extended the Conclusion section, now retitled Conclusion and Future Research as suggested. First, we’ve articulated the theoretical contributions of the study to corpus-based discourse analysis and diplomatic discourse research. Second, we’ve also added a discussion on the practical implications of the findings, including policy-relevant insights for diplomatic communication and national image management. Third, we’ve included a clearer discussion of the broader limitations of the study, particularly regarding data scope, methodological constraints, and generalizability.

Reviewer #3: This paper has potential and does well in positioning alternative approaches to understanding ideology. However, there are some key issues the paper must deal with before it is ready for publication:
(1) clear contribution to the literature. This paper focuses too narrowly on responding to "western media" instead of outlining its clear contributions to the literature, and I think that it has clear contributions to academia that should be laid out. The discussion should explicitly connect the empirical findings to both the contributions and the ideological framework. Could the paper draw on appraisal theory? Soft power theories? or narrative diplomacy/diplomatic rhetoric theories?

Thank you. We’ve revised the Discussion section and the Conclusion section (Conclusion and future research in the revised version as suggested by a reviewer) to highlight this paper’s contributions to the field. Rather than framing the analysis primarily as a response to Western media narratives, we now emphasize how the corpus-based findings contribute to understanding the discursive operationalization of ideology and national image construction in Chinese diplomatic discourse. We’ve also tried to connect the empirical patterns to van Dijk’s Ideological Square, demonstrating how the framework functions dynamically in a Chinese diplomatic context.

Appraisal system and other theories are also relevant to the present study in their emphasis on evaluation and stance-taking. Rather than incorporating multiple theoretical frameworks, we chose to adopt a more closely relative van Dijk’s model. However, following your suggestion, we’ve included a point in the Conclusion section for future studies from other perspectives ‘Future research could also adopt alternative analytical frameworks, such as appraisal theory or narrative approaches, to complement the corpus-based and ideological analysis in this study’.

(2) The methods section does not lay out the methods for reproducibility and rather jumps from data collection to results. What were the preprocessing steps? What were the concerns for segmentation given that this scenario is highly possible with the Chinese corpus? What was the frequency threshold for rare collocates? Where were dispersion or range measures used? What is the reason for choosing MI3 over other association measures? Is it sensitive to results for different window sizes? Why the +5-5 window? What about the operationalisation of attitude, judgement, and affect?

Thank you. In the revised manuscript (p. 11), we’ve added the details of Chinese word segmentation and collocation analysis, including the frequency threshold for collocates, the rationale for the collocational measurement, and the choice of a ±5-word window. It is basically the default setting of WordSmith (8.0).

As for the Appraisal System, we agree that the categorization of attitude, judgement, and affect are highly relevant to the analysis of evaluative meaning in diplomatic discourse. However, adopting appraisal theory as a full analytical framework at this stage would require a fundamentally different coding scheme and analytical focus, which falls beyond the scope of the present study. Instead, we added in the Conclusion section that future studies could be conducted from this perspective.

(3) All counts are raw figures; what about confidence intervals? or chi-square tests for comparing countries? Or bootstrapping or sensitivity for parameter choices?

Thank you. We agree with you that questions of robustness and comparability across countries are very relevant. At the same time, the primary aim of the present study is descriptive, focusing on identifying discursive patterns in a corpus rather than testing probabilistic hypotheses about populations.

Following your suggestion, we’ve clarified in the Method section (p. 12) that the analysis reports raw frequencies and distributional patterns to describe discursive tendencies within the corpus, prioritizing descriptive statistics over inferential testing. In the conclusion part, we’ve also added that inferential statistics such as chi-square tests could be applied in future comparative studies.

(4) The construction of a "national image" is undertheorized. The author's adopt van Dijk's ideological square, but how does it jump from collocates to national image construction? How do the author's categorically code? What does "positive" mean, and how is it represented differently across various discourses? Some words, such as "concern" or "warn", can be considered standard diplomatic language, not hostility, and other discursive shifts may have different meanings. Possibly because of the lack of methodology being laid out, the lack of transparency has me concerned about nuance being lost in the analysis. This should be sufficiently linked back to rhetorical construction as well, and the different rhetorical theories that could be contributing to the construct of "national image".

Thank you. It is true that the notion of “national image” requires clearer theoretical grounding and a more explicit methodological linkage between linguistic evidence and interpretive claims. We’ve revised the manuscript in two ways. First, we’ve clarified how national image is conceptualized in this study as a discursively constructed. Second, we’ve explicitly explained the analytical steps through which collocational patterns are interpreted as indicators of national image construction.

We’ve also clarified that evaluative labels such as “positive” and “negative” do not imply hostility or approval in an absolute sense. Instead, they refer to relative evaluative positioning within a specific diplomatic and discursive context. Terms such as concern or warn were not automatically coded as negative; their evaluative status was determined through contextual and pragmatic analysis of concordance lines.

(5) Provide definitions for conceptual grounding.

Thank you. In response to the previous suggestions, we’ve provided definitions and operational analysis procedures for ideology, national image, and evaluative orientation (positive-negative). We hope that these revisions can enhance the conceptual grounding for our manuscript.

**Overall, we sincerely thank the editor and the reviewers for their constructive and helpful comments, which have substantially improved the quality and clarity of our manuscript.**
