# Supplementary material for: Representing national images of self and others through China’s diplomatic discourse: A corpus-based study
Source: PLoS One. 2026 Jun 12;21(6):e0350726. doi: 10.1371/journal.pone.0350726 (PMC13262881; doi:10.1371/journal.pone.0350726)
Supplement: Attachment — Submitted filename: Response to editors comments.docx [file pone.0350726.s004.docx]

**Response**

We thank all the editor and the reviewers for their critical and constructive comments on the previously revised version of the paper. We’ve revised the paper according to these comments and we believe the paper has now been greatly improved.

**Additional Editor Comments:**

We noticed some potentially unsupported or unclear statements that have not been addressed by the review process. These statements may need clarification, modification, or citations for support. Particularly, the entirety of the second paragraph on page 19,  "Western countries continuously demonized China" and "These actions reflect ongoing efforts by Western countries to maintain their leading positions by exerting pressure on China and hindering its pursuit of rejuvenation." Similarly on page 21 "Faced with the containment and suppression of the US government, China has not shrunk back but responded with a spirit of struggle." (p 21) and "She also expressed that in the face of relentless defamation campaigns by anti-China hostile forces, the spokespersons refused to be silent lambs or gentlemen." (p 21). Additionally, statements on page 26 "condemning their unwanted interference and unwarranted interference" (p 26) In each of these cases are unclear or un supported. These statements need clarfication of being presented as facts, as the authors' opinion, or as the way that these things are depicted by Chinese diplomatic discourses.

Thank you for your constructive suggestions. To follow your feedback, we’ve rewritten the second paragraph on page 19, removed broad and evaluative claims elsewhere, and grounded the discussions in previous research with the support of additional references.

For “the entirety of the second paragraph on page 19,  "Western countries continuously demonized China" and "These actions reflect ongoing efforts by Western countries to maintain their leading positions by exerting pressure on China and hindering its pursuit of rejuvenation. ”, **it has been revised as the following:**

Throughout this period, China was subjected to heightened international scrutiny. A body of scholarship on Western media discourse suggests that some news outlets and political media ecosystems frequently framed China through lenses of blame attribution, including the circulation of racially marked labels such as “Chinese virus” and “Wuhan virus” and related stigmatizing narratives [38-39]. … In this light, such media framings may be understood as aligning with broader Western political efforts to exert pressure on China and to constrain its national rejuvenation agenda [42-43].

For “Similarly on page 21 "Faced with the containment and suppression of the US government, China has not shrunk back but responded with a spirit of struggle." (p 21) and "She also expressed that in the face of relentless defamation campaigns by anti-China hostile forces, the spokespersons refused to be silent lambs or gentlemen." (p 21). ”, **it has been revised as the following:**

In the context of increasingly tense China-US relations, Chinese official discourse has repeatedly emphasized the need to be prepared for “struggle”. For example, in a speech delivered on September 1, 2021 at the opening ceremony of a training session for young officials at the Central Party School, President Xi Jinping stated that, … This viewpoint was echoed by the remarks of Mao Ning, an MFAC spokesperson, during the Third China Spokesperson Forum (May 27, 2023), where Mao highlighted that in the face of relentless defamation campaigns by anti-China hostile forces, the spokespersons refused to be silent lambs or gentlemen but worked as warriors to proactively defend China’s interests, image, and dignity in the fiercely contested international discursive arena.

For “Additionally, statements on page 26 "condemning their unwanted interference and unwarranted interference" (p 26) ”, **it has been revised as the following:**

These offensive strategies involved openly criticizing the ineffective pandemic governance of the US-led western countries and condemning what the MFAC spokesperson, Zhao Lijian, described as hegemonic conducts and unwarranted interference in the internal matters of sovereign nations during the Regular Press Conference on July 12, 2021.

In addition, the 1st paragraph, page 24, describes how Confucianism informs Chinese diplomatic approaches also required a supporting reference.

Thank you for this helpful suggestion. We’ve revised the part and added two pieces of references that examine how Confucian concepts (e.g., yi, li) are connected to China’s strategic culture and contemporary diplomatic discourse.

[Note: HTML markup is below. Please do not edit.]

Reviewers' comments:

Reviewer's Responses to Questions

**Comments to the Author**

1. If the authors have adequately addressed your comments raised in a previous round of review and you feel that this manuscript is now acceptable for publication, you may indicate that here to bypass the “Comments to the Author” section, enter your conflict of interest statement in the “Confidential to Editor” section, and submit your "Accept" recommendation.

Reviewer #1: All comments have been addressed

Reviewer #2: All comments have been addressed

2. Is the manuscript technically sound, and do the data support the conclusions?

The manuscript must describe a technically sound piece of scientific research with data that supports the conclusions. Experiments must have been conducted rigorously, with appropriate controls, replication, and sample sizes. The conclusions must be drawn appropriately based on the data presented.

Reviewer #1: Yes

Reviewer #2: Yes

3. Has the statistical analysis been performed appropriately and rigorously?

Reviewer #1: Yes

Reviewer #2: Yes

4. Have the authors made all data underlying the findings in their manuscript fully available?

The [PLOS Data policy](http://track.editorialmanager.com/CL0/http:%2F%2Fwww.plosone.org%2Fstatic%2Fpolicies.action%23sharing/1/010f019c69a38920-064a76fd-9d8f-4292-8d5e-552166b5b5e1-000000/INSyrK5idCYiqwkOBZE7Gr6x6GnvknjDRxzte7BkTdU=250) requires authors to make all data underlying the findings described in their manuscript fully available without restriction, with rare exception (please refer to the Data Availability Statement in the manuscript PDF file). The data should be provided as part of the manuscript or its supporting information, or deposited to a public repository. For example, in addition to summary statistics, the data points behind means, medians and variance measures should be available. If there are restrictions on publicly sharing data—e.g. participant privacy or use of data from a third party—those must be specified.

Reviewer #1: Yes

Reviewer #2: Yes

5. Is the manuscript presented in an intelligible fashion and written in standard English?

PLOS ONE does not copyedit accepted manuscripts, so the language in submitted articles must be clear, correct, and unambiguous. Any typographical or grammatical errors should be corrected at revision, so please note any specific errors here.

Reviewer #1: Yes

Reviewer #2: Yes

6. Review Comments to the Author

Please use the space provided to explain your answers to the questions above. You may also include additional comments for the author, including concerns about dual publication, research ethics, or publication ethics. (Please upload your review as an attachment if it exceeds 20,000 characters)

Reviewer #1: (No Response)

Reviewer #2: Now this paper can be accepted for publication because authors did a good revision to the manuscript and the paper is much improved.

7. PLOS authors have the option to publish the peer review history of their article ([what does this mean?](https://track.editorialmanager.com/CL0/https:%2F%2Fjournals.plos.org%2Fplosone%2Fs%2Feditorial-and-peer-review-process%23loc-peer-review-history/1/010f019c69a38920-064a76fd-9d8f-4292-8d5e-552166b5b5e1-000000/15H1EnKvoY156bOOevMW5y_zkl4XGf-NfXo3JDaucS8=250)). If published, this will include your full peer review and any attached files.


If you choose “no”, your identity will remain anonymous but your review may still be made public.


**Do you want your identity to be public for this peer review?** For information about this choice, including consent withdrawal, please see our [Privacy Policy](https://track.editorialmanager.com/CL0/https:%2F%2Fwww.plos.org%2Fprivacy-policy/1/010f019c69a38920-064a76fd-9d8f-4292-8d5e-552166b5b5e1-000000/KYUl7FdmcrjOdiNBSoQspA8BxamXp7ZS7JmlK11H5vI=250).

Reviewer #1: No

Reviewer #2: **Yes:** Remus Cretan

**Many thanks again for your constructive comments and suggestions, which helped improve the overall quality of our manuscript.**

**In addition to revising the paper based on the constructive comments and suggestions, we’ve also carefully reviewed the manuscript and made a few other minor revisions where we found necessary.**

[NOTE: If reviewer comments were submitted as an attachment file, they will be attached to this email and accessible via the submission site. Please log into your account, locate the manuscript record, and check for the action link "View Attachments". If this link does not appear, there are no attachment files.]

To ensure your figures meet our technical requirements, please review our figure guidelines: https://journals.plos.org/plosone/s/figures

You may also use PLOS’s free figure tool, NAAS, to help you prepare publication quality figures: https://journals.plos.org/plosone/s/figures#loc-tools-for-figure-preparation.

NAAS will assess whether your figures meet our technical requirements by comparing each figure against our figure specifications.
