# Supplementary material for: Representing national images of self and others through China’s diplomatic discourse: A corpus-based study
Source: PLoS One. 2026 Jun 12;21(6):e0350726. doi: 10.1371/journal.pone.0350726 (PMC13262881; doi:10.1371/journal.pone.0350726)
Supplement: Peer review history

# Peer review: Decision Letter 0

**DOI:** 10.1371/journal.pone.0350726.r001

**Authors:** Muhammad Khalil Khan

24 Nov 2025

PONE-D-25-50168

Representing national images of Self and Others through China’s Diplomatic discourse

PLOS ONE

Dear Dr. Li,

Thank you for submitting your manuscript to PLOS ONE. After careful consideration, we feel that it has merit but does not fully meet PLOS ONE’s publication criteria as it currently stands. Therefore, we invite you to submit a revised version of the manuscript that addresses the points raised during the review process.

Please submit your revised manuscript by Jan 02 2026 11:59PM. If you will need more time than this to complete your revisions, please reply to this message or contact the journal office at plosone@plos.org. When you're ready to submit your revision, log on to https://www.editorialmanager.com/pone/ and select the 'Submissions Needing Revision' folder to locate your manuscript file.

Please include the following items when submitting your revised manuscript:

- A rebuttal letter that responds to each point raised by the academic editor and reviewer(s). You should upload this letter as a separate file labeled 'Response to Reviewers'.
- A marked-up copy of your manuscript that highlights changes made to the original version. You should upload this as a separate file labeled 'Revised Manuscript with Track Changes'.
- An unmarked version of your revised paper without tracked changes. You should upload this as a separate file labeled 'Manuscript'.

If you would like to make changes to your financial disclosure, please include your updated statement in your cover letter. Guidelines for resubmitting your figure files are available below the reviewer comments at the end of this letter.

If applicable, we recommend that you deposit your laboratory protocols in protocols.io to enhance the reproducibility of your results. Protocols.io assigns your protocol its own identifier (DOI) so that it can be cited independently in the future. For instructions see: https://journals.plos.org/plosone/s/submission-guidelines#loc-laboratory-protocols. Additionally, PLOS ONE offers an option for publishing peer-reviewed Lab Protocol articles, which describe protocols hosted on protocols.io. Read more information on sharing protocols at https://plos.org/protocols?utm_medium=editorial-email&utm_source=authorletters&utm_campaign=protocols.

We look forward to receiving your revised manuscript.

Kind regards,

Muhammad Khalil Khan

Academic Editor

PLOS ONE

Journal Requirements:

When submitting your revision, we need you to address these additional requirements.

1. Please ensure that your manuscript meets PLOS ONE's style requirements, including those for file naming. The PLOS ONE style templates can be found at

https://journals.plos.org/plosone/s/file?id=wjVg/PLOSOne_formatting_sample_main_body.pdf and

https://journals.plos.org/plosone/s/file?id=ba62/PLOSOne_formatting_sample_title_authors_affiliations.pdf

2. We note that the grant information you provided in the ‘Funding Information’ and ‘Financial Disclosure’ sections do not match.

When you resubmit, please ensure that you provide the correct grant numbers for the awards you received for your study in the ‘Funding Information’ section.

3. Thank you for stating the following financial disclosure:

“This is part of the Research Project of The National Social Science Fund of China (24BYY147), Humanities and Social Sciences of the Ministry of Education (23YJA740017), Shanghai Pujiang Talent Program (24PJC045).”

Please state what role the funders took in the study.  If the funders had no role, please state: "The funders had no role in study design, data collection and analysis, decision to publish, or preparation of the manuscript." If this statement is not correct you must amend it as needed.

Please include this amended Role of Funder statement in your cover letter; we will change the online submission form on your behalf.

4. Please note that funding information should not appear in the Acknowledgments section or other areas of your manuscript. We will only publish funding information present in the Funding Statement section of the online submission form. Please remove any funding-related text from the manuscript.

5. Please note that your Data Availability Statement is currently missing the repository name. If your manuscript is accepted for publication, you will be asked to provide these details on a very short timeline. We therefore suggest that you provide this information now, though we will not hold up the peer review process if you are unable.

6. Please amend either the title on the online submission form (via Edit Submission) or the title in the manuscript so that they are identical.

7.  We note you have included a table to which you do not refer in the text of your manuscript. Please ensure that you refer to Table 4 in your text; if accepted, production will need this reference to link the reader to the Table.

8. If the reviewer comments include a recommendation to cite specific previously published works, please review and evaluate these publications to determine whether they are relevant and should be cited. There is no requirement to cite these works unless the editor has indicated otherwise.

**Additional Editor Comments:**

All three reviewers find you manuscript interesting but suggested some major revision before further porcessing. Therefore, I decided to provide you with an opportunity to revise and resubmit you manscript according.  Reviewers comments are attached with email.

[Note: HTML markup is below. Please do not edit.]

Reviewers' comments:

Reviewer's Responses to Questions

**Comments to the Author**

1. Is the manuscript technically sound, and do the data support the conclusions?

The manuscript must describe a technically sound piece of scientific research with data that supports the conclusions. Experiments must have been conducted rigorously, with appropriate controls, replication, and sample sizes. The conclusions must be drawn appropriately based on the data presented.

Reviewer #1: Partly

Reviewer #2: Yes

Reviewer #3: No

**********

2. Has the statistical analysis been performed appropriately and rigorously?

Reviewer #1: Yes

Reviewer #2: N/A

Reviewer #3: No

**********

3. Have the authors made all data underlying the findings in their manuscript fully available?

The PLOS Data policy requires authors to make all data underlying the findings described in their manuscript fully available without restriction, with rare exception (please refer to the Data Availability Statement in the manuscript PDF file). The data should be provided as part of the manuscript or its supporting information, or deposited to a public repository. For example, in addition to summary statistics, the data points behind means, medians and variance measures should be available. If there are restrictions on publicly sharing data—e.g. participant privacy or use of data from a third party—those must be specified.

Reviewer #1: Yes

Reviewer #2: Yes

Reviewer #3: Yes

**********

4. Is the manuscript presented in an intelligible fashion and written in standard English?

PLOS ONE does not copyedit accepted manuscripts, so the language in submitted articles must be clear, correct, and unambiguous. Any typographical or grammatical errors should be corrected at revision, so please note any specific errors here.

Reviewer #1: No

Reviewer #2: Yes

Reviewer #3: Yes

**********

5. Review Comments to the Author

Please use the space provided to explain your answers to the questions above. You may also include additional comments for the author, including concerns about dual publication, research ethics, or publication ethics. (Please upload your review as an attachment if it exceeds 20,000 characters)

Reviewer #1: Peer Review Report

The study “Representing national images of Self and Others through China’s Diplomatic Discourse: A Corpus-based Study” is timely and relevant, as it addresses the interplay between discourse, ideology, and national image in Chinese diplomacy. It has a Solid methodological framework that combines corpus linguistics with critical discourse analysis using van Dijk’s Ideological Square as a theoretical foundation. However, many areas need to be addressed.

Comments:

1. Abstract is dense, lacks methods, and key contributions.

2. Theoretical framework relies heavily on van Dijk without integrating newer discourse theories.

3. Discussion occasionally leans toward political commentary rather than data-based interpretation.

4. The role of Confucian philosophy needs a stronger textual linkage to corpus evidence.

5. Language requires minor proofreading to correct article use and reduce redundancy.

Suggestions:

1. Simplify and clarify the Abstract; include corpus details and specify the study’s unique contribution.

2. Clarify the research gap in the Introduction and define 'Self' and 'Other' earlier.

3. Integrate more recent works in critical discourse studies (e.g., multimodal CDA, framing theory).

4. Add coder reliability metrics such as Cohen’s Kappa to enhance methodological transparency.

5. Include a brief justification for choosing the five UNSC countries and discuss implications for generalizability.

6. Ensure Discussion links empirical results directly to ideological interpretation rather than general political commentary.

7. The manuscript would benefit from clearer structural organization. Currently, several sections blend conceptually, making it difficult to distinguish between major components. The Introduction (pp. 4–6) merges literature review material, which should be moved to a separate section titled 'Literature Review' following the Introduction. Similarly, 'Research Design', 'Method', and 'Data Collection' (pp. 7–10) overlap and should be consolidated under a unified 'Methodology' section with subsections such as 'Research Design and Rationale', 'Data Collection and Corpus Construction', and 'Analytical Procedures'. The Results and Discussion (pp. 10–21) intermingle; interpretive commentary should be reserved for the Discussion to maintain analytical clarity. The Conclusion (pp. 22–23) could be reframed as 'Conclusion and Future Research' to emphasize a forward-looking approach.

8. Conclude with theoretical contributions to corpus-based discourse analysis and future comparative research directions.

9. Add a dedicated Limitations subsection addressing corpus scope and exclusion of journalists’ questions.

10. Finally, the manuscript requires careful proofreading to address several minor but recurring issues of grammar, punctuation, and citation style. For example, on pages 6–7, in-text citations such as “van Dijk [19, 267; 20]; [21, 128]” are inconsistently punctuated and formatted. Similarly, within the same section, numbered items in the Ideological Square model— (1) Express/emphasize information that is positive about Us; … (4) Suppress/de-emphasize information that is negative about Us”—contain inconsistent capitalization and spacing around conjunctions (“Us”, “Them”). On page 8, the citation of external web resources— “These transcripts are available at https://www.fmprc.gov.cn/eng/xw/fyrbt/”—should follow standard referencing conventions, ideally formatted as a reference entry or footnote rather than embedded in the running text.

Need major revisions.

Reviewer #2: This is an interesting paper on representing national images of self and others through China’s Diplomatic discourse. The study is based on discursive analysis of spokemen for China’s Ministry of Foreign Affairs during regular press conferences and shows how these discursive strategies shape the national images of China and the other four permanent members of the United Nations Security Council. The results are good and highlight China’s stance and international cooperation initiatives while also using offensive discourse strategy to counter criticisms from US-led Western nations and media regarding the pandemic period as well as some positive self-presentation and negative other-presentation to further justify the rationality of China’s stance and promote China’s image.

Besides its merits, some revisions are needed throughout the paper.

First, the introduction and the conceptual background of the paper should be more grounded in international diplomatic studies and this is also reflected in the scarce reference sources of this paper (32 references are few for a good academic paper!).

Second, the method is anchored in discourse analysis and authors present the advantages of this method but readers need to know more about the limits of the method and data and what were the challenges in collecting those data. More details on how data was interpreted need to be addressed.

Third, the discussions related to the results of the paper should be more related to international diplomatic studies.

Finally, the conclusions are too short. They should also include not only the theoretical perspectives but also the practical dimensions of the results, so some policy recommendations should be mentioned. Also, the broader limitation of the study has to be mentioned in the conclusions.

Reviewer #3: This paper has potential and does well in positioning alternative approaches to understanding ideology. However, there are some key issues the paper must deal with before it is ready for publication:

(1) clear contribution to the literature. This paper focuses too narrowly on responding to "western media" instead of outlining its clear contributions to the literature, and I think that it has clear contributions to academia that should be laid out. The discussion should explicitly connect the empirical findings to both the contributions and the ideological framework. Could the paper draw on appraisal theory? Soft power theories? or narrative diplomacy/diplomatic rhetoric theories?

(2) The methods section does not lay out the methods for reproducibility and rather jumps from data collection to results. What were the preprocessing steps? What were the concerns for segmentation given that this scenario is highly possible with the Chinese corpus? What was the frequency threshold for rare collocates? Where were dispersion or range measures used? What is the reason for choosing MI3 over other association measures? Is it sensitive to results for different window sizes? Why the +5-5 window? What about the operationalisation of attitude, judgement, and affect?

(3) All counts are raw figures; what about confidence intervals? or chi-square tests for comparing countries? Or bootstrapping or sensitivity for parameter choices?

(4) The construction of a "national image" is undertheorized. The author's adopt van Dijk's ideological square, but how does it jump from collocates to national image construction? How do the author's categorically code? What does "positive" mean, and how is it represented differently across various discourses? Some words, such as "concern" or "warn", can be considered standard diplomatic language, not hostility, and other discursive shifts may have different meanings. Possibly because of the lack of methodology being laid out, the lack of transparency has me concerned about nuance being lost in the analysis. This should be sufficiently linked back to rhetorical construction as well, and the different rhetorical theories that could be contributing to the construct of "national image".

(5) Provide definitions for conceptual grounding.

**********

6. PLOS authors have the option to publish the peer review history of their article (what does this mean?). If published, this will include your full peer review and any attached files.

If you choose “no”, your identity will remain anonymous but your review may still be made public.

**Do you want your identity to be public for this peer review?**  For information about this choice, including consent withdrawal, please see our Privacy Policy.

Reviewer #1: **Yes:**  Dharm Dev Bhatta

Reviewer #2: No

Reviewer #3: No

**********

[NOTE: If reviewer comments were submitted as an attachment file, they will be attached to this email and accessible via the submission site. Please log into your account, locate the manuscript record, and check for the action link "View Attachments". If this link does not appear, there are no attachment files.]

To ensure your figures meet our technical requirements, please review our figure guidelines: https://journals.plos.org/plosone/s/figures

You may also use PLOS’s free figure tool, NAAS, to help you prepare publication quality figures: https://journals.plos.org/plosone/s/figures#loc-tools-for-figure-preparation.

NAAS will assess whether your figures meet our technical requirements by comparing each figure against our figure specifications.

Attachment Submitted filename: Peer_Review_PLOS_ONE_China_Diplomatic_Discourse.docx

# Author comment: Author response to Decision Letter 1

**DOI:** 10.1371/journal.pone.0350726.r002

12 Jan 2026

Journal Requirements:

When submitting your revision, we need you to address these additional requirements.

1. Please ensure that your manuscript meets PLOS ONE's style requirements, including those for file naming. The PLOS ONE style templates can be found at

https://journals.plos.org/plosone/s/file?id=wjVg/PLOSOne_formatting_sample_main_body.pdf and https://journals.plos.org/plosone/s/file?id=ba62/PLOSOne_formatting_sample_title_authors_affiliations.pdf

Thank you. We have revised the formats accordingly.

2. We note that the grant information you provided in the ‘Funding Information’ and ‘Financial Disclosure’ sections do not match.

When you resubmit, please ensure that you provide the correct grant numbers for the awards you received for your study in the ‘Funding Information’ section.

Thank you for pointing it out.

3. Thank you for stating the following financial disclosure:

“This is part of the Research Projects of The National Social Science Fund of China (20&ZD140), Humanities and Social Sciences of the Ministry of Education (23YJA740017), Shanghai Pujiang Talent Program (24PJC045).”

Please state what role the funders took in the study. If the funders had no role, please state: "The funders had no role in study design, data collection and analysis, decision to publish, or preparation of the manuscript." If this statement is not correct you must amend it as needed.

Please include this amended Role of Funder statement in your cover letter; we will change the online submission form on your behalf.

Thank you. The corresponding author, Tao Li, is the CI of the Major Project of The National Social Science Fund of China (20&ZD140), and the PI for the other two projects, i.e., Humanities and Social Sciences of the Ministry of Education (23YJA740017), Shanghai Pujiang Talent Program (24PJC045).

4. Please note that funding information should not appear in the Acknowledgments section or other areas of your manuscript. We will only publish funding information present in the Funding Statement section of the online submission form. Please remove any funding-related text from the manuscript.

Thank you for your reminder. We’ve removed it.

5. Please note that your Data Availability Statement is currently missing the repository name. If your manuscript is accepted for publication, you will be asked to provide these details on a very short timeline. We therefore suggest that you provide this information now, though we will not hold up the peer review process if you are unable.

Thank you. Just checked. It is still working.

https://github.com/lt0806/Data/blob/main/MFAC_Data-main.zip

6. Please amend either the title on the online submission form (via Edit Submission) or the title in the manuscript so that they are identical.

Thank you for your reminder.

7. We note you have included a table to which you do not refer in the text of your manuscript. Please ensure that you refer to Table 4 in your text; if accepted, production will need this reference to link the reader to the Table.

Thank you very much for pointing it out. We’ve deleted two tables which we found not necessary in the previous versions but forgot to change others. Now they are changed.

8. If the reviewer comments include a recommendation to cite specific previously published works, please review and evaluate these publications to determine whether they are relevant and should be cited. There is no requirement to cite these works unless the editor has indicated otherwise.

Thank you for your reminder.

Additional Editor Comments:

All three reviewers find you manuscript interesting but suggested some major revision before further porcessing. Therefore, I decided to provide you with an opportunity to revise and resubmit you manscript according. Reviewers comments are attached with email.

Thank you very much. We’ve revised our manuscript in accordance with your and the reviewers’ suggestions and comments.

Review Comments to the Author

Reviewer #1: Peer Review Report

The study “Representing national images of Self and Others through China’s Diplomatic Discourse: A Corpus-based Study” is timely and relevant, as it addresses the interplay between discourse, ideology, and national image in Chinese diplomacy. It has a Solid methodological framework that combines corpus linguistics with critical discourse analysis using van Dijk’s Ideological Square as a theoretical foundation. However, many areas need to be addressed.

Comments:

1. Abstract is dense, lacks methods, and key contributions.

Thank you. I’ve added methods and contributions.

2. Theoretical framework relies heavily on van Dijk without integrating newer discourse theories.

Thank you. We’ve added reviews of the new frameworks of discourse analysis as you can see on pages 6-8. We’ve also included these new citations in the reference part.

3. Discussion occasionally leans toward political commentary rather than data-based interpretation.

Thank you. We’ve revised some parts of it to make the discussion more related to the data. Details can be seen in the whole discussion section.

4. The role of Confucian philosophy needs a stronger textual linkage to corpus evidence.

Thank you. We’ve revised it to improve the connections between the two.

5. Language requires minor proofreading to correct article use and reduce redundancy.

Suggestions:

1. Simplify and clarify the Abstract; include corpus details and specify the study’s unique contribution.

Thank you. We’ve revised it as you suggested above.

2. Clarify the research gap in the Introduction and define 'Self' and 'Other' earlier.

Thank you. We’ve revised it accordingly.

3. Integrate more recent works in critical discourse studies (e.g., multimodal CDA, framing theory).

Thank you. We’ve added some reviews of the new frameworks of discourse analysis as you can see on pages 6-8. We’ve also included these new citations in the reference part.

4. Add coder reliability metrics such as Cohen’s Kappa to enhance methodological transparency.

Thank you. It is true that it might increase reliability of the results. We didn’t use interrater reliability test because we chose only those two coders shared. Anyway, we’ve included this limitation in the conclusion section.

5. Include a brief justification for choosing the five UNSC countries and discuss implications for generalizability.

Thank you. We’ve added a brief justification for choosing the five countries as you can see on page 11. We acknowledge that focusing on the five countries limits the scope of generalizability, which is also included in the conclusion section.

6. Ensure Discussion links empirical results directly to ideological interpretation rather than general political commentary.

Thank you. We’ve revised some parts of it to make the discussion more related to the data. Details can be seen in the whole discussion section.

7. The manuscript would benefit from clearer structural organization. Currently, several sections blend conceptually, making it difficult to distinguish between major components. The Introduction (pp. 4–6) merges literature review material, which should be moved to a separate section titled 'Literature Review' following the Introduction. Similarly, 'Research Design', 'Method', and 'Data Collection' (pp. 7–10) overlap and should be consolidated under a unified 'Methodology' section with subsections such as 'Research Design and Rationale', 'Data Collection and Corpus Construction', and 'Analytical Procedures'. The Results and Discussion (pp. 10–21) intermingle; interpretive commentary should be reserved for the Discussion to maintain analytical clarity. The Conclusion (pp. 22–23) could be reframed as 'Conclusion and Future Research' to emphasize a forward-looking approach.

Thank you. We agree that clearer structure is important for reader accessibility and we’ve revised the manuscript accordingly.

8. Conclude with theoretical contributions to corpus-based discourse analysis and future comparative research directions.

Thank you. We’ve added it as you can see in the conclusion.

9. Add a dedicated Limitations subsection addressing corpus scope and exclusion of journalists’ questions.

Thank you. We’ve added limitations in the conclusion.

10. Finally, the manuscript requires careful proofreading to address several minor but recurring issues of grammar, punctuation, and citation style. For example, on pages 6–7, in-text citations such as “van Dijk [19, 267; 20]; [21, 128]” are inconsistently punctuated and formatted. Similarly, within the same section, numbered items in the Ideological Square model— (1) Express/emphasize information that is positive about Us; … (4) Suppress/de-emphasize information that is negative about Us”—contain inconsistent capitalization and spacing around conjunctions (“Us”, “Them”). On page 8, the citation of external web resources— “These transcripts are available at https://www.fmprc.gov.cn/eng/xw/fyrbt/”—should follow standard referencing conventions, ideally formatted as a reference entry or footnote rather than embedded in the running text.

Thank you. We’ve read through the whole manuscript and revised the formats accordingly. In “van Dijk [19, 267; 20]”, “19” and “20” refer to different citations with “;” to separate them and “267” is page number. 19 is a monograph while 20 is a journal paper which doesn’t require a page number. However, there is one exception about the external web resource without an author. It seems there is no such rule for this kind of reference in PlosOne’s requirement and we don’t know how to deal with it.

Overall, we are very grateful for your detailed and constructive suggestions, which have largely improved the clarity and quality of our manuscript.

Reviewer #2: This is an interesting paper on representing national images of self and others through China’s Diplomatic discourse. The study is based on discursive analysis of spokemen for China’s Ministry of Foreign Affairs during regular press conferences and shows how these discursive strategies shape the national images of China and the other four permanent members of the United Nations Security Council. The results are good and highlight China’s stance and international cooperation initiatives while also using offensive discourse strategy to counter criticisms from US-led Western nations and media regarding the pandemic period as well as some positive self-presentation and negative other-presentation to further justify the rationality of China’s stance and promote China’s image.

Thank you for your positive comments.

Besides its merits, some revisions are needed throughout the paper.

First, the introduction and the conceptual background of the paper should be more grounded in international diplomatic studies and this is also reflected in the scarce reference sources of this paper (32 references are few for a good academic paper!).

Thank you. Following your suggestion, we’ve added a background to strengthen its engagement with relevant scholarship in international diplomatic studies and crisis communication. We also included in the discussion part more engagement with international diplomatic studies. As a result, the reference list has been enriched as well.

Second, the method is anchored in discourse analysis and authors present the advantages of this method but readers need to know more about the limits of the method and data and what were the challenges in collecting those data. More details on how data was interpreted need to be addressed.

Thank you for this important methodological comment. We’ve revised the Methodology section accordingly. We’ve included a paragraph in Methodology and another one in Conclusion section as well, discussing the issues related to institutional genre constraints, the scope of generalizability, and how corpus findings were interpreted by combining quantitative patterns with close qualitative reading.

Third, the discussions related to the results of the paper should be more related to international diplomatic studies.

Thank you. We’ve rewritten the Discussion section and included more discussions in the scope of international diplomatic studies with revisions made particularly on pages from 18 to 24.

Finally, the conclusions are too short. They should also include not only the theoretical perspectives but also the practical dimensions of the results, so some policy recommendations should be mentioned. Also, the broader limitation of the study has to be mentioned in the conclusions.

Thank you for this valuable suggestion. We’ve revised and extended the Conclusion section, now retitled Conclusion and Future Research as suggested. First, we’ve articulated the theoretical contributions of the study to corpus-based discourse analysis and diplomatic discourse research. Second, we’ve also added a discussion on the practical implications of the findings, including policy-relevant insights for diplomatic communication and national image management. Third, we’ve included a clearer discussion of the broader limitations of the study, particularly regarding data scope, methodological constraints, and generalizability.

Reviewer #3: This paper has potential and does well in positioning alternative approaches to understanding ideology. However, there are some key issues the paper must deal with before it is ready for publication:

(1) clear contribution to the literature. This paper focuses too narrowly on responding to "western media" instead of outlining its clear contributions to the literature, and I think that it has clear contributions to academia that should be laid out. The discussion should explicitly connect the empirical findings to both the contributions and the ideological framework. Could the paper draw on appraisal theory? Soft power theories? or narrative diplomacy/diplomatic rhetoric theories?

Thank you. We’ve revised the Discussion section and the Conclusion section (Conclusion and future research in the revised version as suggested by a reviewer) to highlight this paper’s contributions to the field. Rather than framing the analysis primarily as a response to Western media narratives, we now emphasize how the corpus-based findings contribute to understanding the discursive operationalization of ideology and national image construction in Chinese diplomatic discourse. We’ve also tried to connect the empirical patterns to van Dijk’s Ideological Square, demonstrating how the framework functions dynamically in a Chinese diplomatic context.

Appraisal system and other theories are also relevant to the present study in their emphasis on evaluation and stance-taking. Rather than incorporating multiple theoretical frameworks, we chose to adopt a more closely relative van Dijk’s model. However, following your suggestion, we’ve included a point in the Conclusion section for future studies from other perspectives ‘Future research could also adopt alternative analytical frameworks, such as appraisal theory or narrative approaches, to complement the corpus-based and ideological analysis in this study’.

(2) The methods section does not lay out the methods for reproducibility and rather jumps from data collection to results. What were the preprocessing steps? What were the concerns for segmentation given that this scenario is highly possible with the Chinese corpus? What was the frequency threshold for rare collocates? Where were dispersion or range measures used? What is the reason for choosing MI3 over other association measures? Is it sensitive to results for different window sizes? Why the +5-5 window? What about the operationalisation of attitude, judgement, and affect?

Thank you. In the revised manuscript (p. 11), we’ve added the details of Chinese word segmentation and collocation analysis, including the frequency threshold for collocates, the rationale for the collocational measurement, and the choice of a ±5-word window. It is basically the default setting of WordSmith (8.0).

As for the Appraisal System, we agree that the categorization of attitude, judgement, and affect are highly relevant to the analysis of evaluative meaning in diplomatic discourse. However, adopting appraisal theory as a full analytical framework at this stage would require a fundamentally different coding s

Attachment Submitted filename: 1.9 Response to Reviewers.docx

# Peer review: Decision Letter 1

**DOI:** 10.1371/journal.pone.0350726.r003

**Authors:** Muhammad Khalil Khan

16 Feb 2026

PONE-D-25-50168R1

Representing national images of Self and Others through China’s diplomatic discourse: A corpus-based study

PLOS One

Dear Dr. Li,

Thank you for submitting your manuscript to PLOS ONE. After careful consideration, we feel that it has merit but does not fully meet PLOS ONE’s publication criteria as it currently stands. Therefore, we invite you to submit a revised version of the manuscript that addresses the points raised during the review process.

Please submit your revised manuscript by Apr 02 2026 11:59PM. If you will need more time than this to complete your revisions, please reply to this message or contact the journal office at plosone@plos.org. When you're ready to submit your revision, log on to https://www.editorialmanager.com/pone/ and select the 'Submissions Needing Revision' folder to locate your manuscript file.

Please include the following items when submitting your revised manuscript:

- A letter that responds to each point raised by the academic editor and reviewer(s). You should upload this letter as a separate file labeled 'Response to Reviewers'.
- A marked-up copy of your manuscript that highlights changes made to the original version. You should upload this as a separate file labeled 'Revised Manuscript with Track Changes'.
- An unmarked version of your revised paper without tracked changes. You should upload this as a separate file labeled 'Manuscript'.

If you would like to make changes to your financial disclosure, please include your updated statement in your cover letter. Guidelines for resubmitting your figure files are available below the reviewer comments at the end of this letter.

If applicable, we recommend that you deposit your laboratory protocols in protocols.io to enhance the reproducibility of your results. Protocols.io assigns your protocol its own identifier (DOI) so that it can be cited independently in the future. For instructions see: https://journals.plos.org/plosone/s/submission-guidelines#loc-laboratory-protocols. Additionally, PLOS ONE offers an option for publishing peer-reviewed Lab Protocol articles, which describe protocols hosted on protocols.io. Read more information on sharing protocols at https://plos.org/protocols?utm_medium=editorial-email&utm_source=authorletters&utm_campaign=protocols.

We look forward to receiving your revised manuscript.

Kind regards,

Muhammad Khalil Khan

Academic Editor

PLOS One

Journal Requirements:

If the reviewer comments include a recommendation to cite specific previously published works, please review and evaluate these publications to determine whether they are relevant and should be cited. There is no requirement to cite these works unless the editor has indicated otherwise.

Please review your reference list to ensure that it is complete and correct. If you have cited papers that have been retracted, please include the rationale for doing so in the manuscript text, or remove these references and replace them with relevant current references. Any changes to the reference list should be mentioned in the rebuttal letter that accompanies your revised manuscript. If you need to cite a retracted article, indicate the article’s retracted status in the References list and also include a citation and full reference for the retraction notice.

**Additional Editor Comments:**

We noticed some potentially unsupported or unclear statements that have not been addressed by the review process. These statements may need clarification, modification, or citations for support. Particularly,  the entirety of the second paragraph on page 19,  "Western countries continuously demonized China" and "These actions reflect ongoing efforts by Western countries to maintain their leading positions by exerting pressure on China and hindering its pursuit of rejuvenation." Similarly on page 21 "Faced with the containment and suppression of the US government, China has not shrunk back but responded with a spirit of struggle." (p 21) and "She also expressed that in the face of relentless defamation campaigns by anti-China hostile forces, the spokespersons refused to be silent lambs or gentlemen." (p 21). Additionally, statements on page 26 "condemning their unwanted interference and unwarranted interference" (p 26)

In each of these cases are unclear or un supported. These statements need clarfication of being presented as facts, as the authors' opinion, or as the way that these things are depicted by Chinese diplomatic discourses. In addition, the 1st paragraph, page 24, describes how Confucianism informs Chinese diplomatic approaches also required a supporting reference.

[Note: HTML markup is below. Please do not edit.]

Reviewers' comments:

Reviewer's Responses to Questions

**Comments to the Author**

1. If the authors have adequately addressed your comments raised in a previous round of review and you feel that this manuscript is now acceptable for publication, you may indicate that here to bypass the “Comments to the Author” section, enter your conflict of interest statement in the “Confidential to Editor” section, and submit your "Accept" recommendation.

Reviewer #1: All comments have been addressed

Reviewer #2: All comments have been addressed

**********

2. Is the manuscript technically sound, and do the data support the conclusions?

The manuscript must describe a technically sound piece of scientific research with data that supports the conclusions. Experiments must have been conducted rigorously, with appropriate controls, replication, and sample sizes. The conclusions must be drawn appropriately based on the data presented.

Reviewer #1: Yes

Reviewer #2: Yes

**********

3. Has the statistical analysis been performed appropriately and rigorously?

Reviewer #1: Yes

Reviewer #2: Yes

**********

4. Have the authors made all data underlying the findings in their manuscript fully available?

The PLOS Data policy requires authors to make all data underlying the findings described in their manuscript fully available without restriction, with rare exception (please refer to the Data Availability Statement in the manuscript PDF file). The data should be provided as part of the manuscript or its supporting information, or deposited to a public repository. For example, in addition to summary statistics, the data points behind means, medians and variance measures should be available. If there are restrictions on publicly sharing data—e.g. participant privacy or use of data from a third party—those must be specified.

Reviewer #1: Yes

Reviewer #2: Yes

**********

5. Is the manuscript presented in an intelligible fashion and written in standard English?

PLOS ONE does not copyedit accepted manuscripts, so the language in submitted articles must be clear, correct, and unambiguous. Any typographical or grammatical errors should be corrected at revision, so please note any specific errors here.

Reviewer #1: Yes

Reviewer #2: Yes

**********

6. Review Comments to the Author

Please use the space provided to explain your answers to the questions above. You may also include additional comments for the author, including concerns about dual publication, research ethics, or publication ethics. (Please upload your review as an attachment if it exceeds 20,000 characters)

Reviewer #1: (No Response)

Reviewer #2: Now this paper can be accepted for publication because authors did a good revision to the manuscript and the paper is much improved.

**********

7. PLOS authors have the option to publish the peer review history of their article (what does this mean?). If published, this will include your full peer review and any attached files.

If you choose “no”, your identity will remain anonymous but your review may still be made public.

**Do you want your identity to be public for this peer review?**  For information about this choice, including consent withdrawal, please see our Privacy Policy.

Reviewer #1: No

Reviewer #2: **Yes:**  Remus Cretan

**********

[NOTE: If reviewer comments were submitted as an attachment file, they will be attached to this email and accessible via the submission site. Please log into your account, locate the manuscript record, and check for the action link "View Attachments". If this link does not appear, there are no attachment files.]

To ensure your figures meet our technical requirements, please review our figure guidelines: https://journals.plos.org/plosone/s/figures

You may also use PLOS’s free figure tool, NAAS, to help you prepare publication quality figures: https://journals.plos.org/plosone/s/figures#loc-tools-for-figure-preparation.

NAAS will assess whether your figures meet our technical requirements by comparing each figure against our figure specifications.

# Author comment: Author response to Decision Letter 2

**DOI:** 10.1371/journal.pone.0350726.r004

16 Mar 2026

Response

We thank all the editor and the reviewers for their critical and constructive comments on the previously revised version of the paper. We’ve revised the paper according to these comments and we believe the paper has now been greatly improved.

Additional Editor Comments:

We noticed some potentially unsupported or unclear statements that have not been addressed by the review process. These statements may need clarification, modification, or citations for support. Particularly, the entirety of the second paragraph on page 19, "Western countries continuously demonized China" and "These actions reflect ongoing efforts by Western countries to maintain their leading positions by exerting pressure on China and hindering its pursuit of rejuvenation." Similarly on page 21 "Faced with the containment and suppression of the US government, China has not shrunk back but responded with a spirit of struggle." (p 21) and "She also expressed that in the face of relentless defamation campaigns by anti-China hostile forces, the spokespersons refused to be silent lambs or gentlemen." (p 21). Additionally, statements on page 26 "condemning their unwanted interference and unwarranted interference" (p 26) In each of these cases are unclear or un supported. These statements need clarfication of being presented as facts, as the authors' opinion, or as the way that these things are depicted by Chinese diplomatic discourses.

Thank you for your constructive suggestions. To follow your feedback, we’ve rewritten the second paragraph on page 19, removed broad and evaluative claims elsewhere, and grounded the discussions in previous research with the support of additional references.

For “the entirety of the second paragraph on page 19, "Western countries continuously demonized China" and "These actions reflect ongoing efforts by Western countries to maintain their leading positions by exerting pressure on China and hindering its pursuit of rejuvenation. ”, it has been revised as the following:

Throughout this period, China was subjected to heightened international scrutiny. A body of scholarship on Western media discourse suggests that some news outlets and political media ecosystems frequently framed China through lenses of blame attribution, including the circulation of racially marked labels such as “Chinese virus” and “Wuhan virus” and related stigmatizing narratives [38-39]. … In this light, such media framings may be understood as aligning with broader Western political efforts to exert pressure on China and to constrain its national rejuvenation agenda [42-43].

For “Similarly on page 21 "Faced with the containment and suppression of the US government, China has not shrunk back but responded with a spirit of struggle." (p 21) and "She also expressed that in the face of relentless defamation campaigns by anti-China hostile forces, the spokespersons refused to be silent lambs or gentlemen." (p 21). ”, it has been revised as the following:

In the context of increasingly tense China-US relations, Chinese official discourse has repeatedly emphasized the need to be prepared for “struggle”. For example, in a speech delivered on September 1, 2021 at the opening ceremony of a training session for young officials at the Central Party School, President Xi Jinping stated that, … This viewpoint was echoed by the remarks of Mao Ning, an MFAC spokesperson, during the Third China Spokesperson Forum (May 27, 2023), where Mao highlighted that in the face of relentless defamation campaigns by anti-China hostile forces, the spokespersons refused to be silent lambs or gentlemen but worked as warriors to proactively defend China’s interests, image, and dignity in the fiercely contested international discursive arena.

For “Additionally, statements on page 26 "condemning their unwanted interference and unwarranted interference" (p 26) ”, it has been revised as the following:

These offensive strategies involved openly criticizing the ineffective pandemic governance of the US-led western countries and condemning what the MFAC spokesperson, Zhao Lijian, described as hegemonic conducts and unwarranted interference in the internal matters of sovereign nations during the Regular Press Conference on July 12, 2021.

In addition, the 1st paragraph, page 24, describes how Confucianism informs Chinese diplomatic approaches also required a supporting reference.

Thank you for this helpful suggestion. We’ve revised the part and added two pieces of references that examine how Confucian concepts (e.g., yi, li) are connected to China’s strategic culture and contemporary diplomatic discourse.

[Note: HTML markup is below. Please do not edit.]

Reviewers' comments:

Reviewer's Responses to Questions

Comments to the Author

1. If the authors have adequately addressed your comments raised in a previous round of review and you feel that this manuscript is now acceptable for publication, you may indicate that here to bypass the “Comments to the Author” section, enter your conflict of interest statement in the “Confidential to Editor” section, and submit your "Accept" recommendation.

Reviewer #1: All comments have been addressed

Reviewer #2: All comments have been addressed

________________________________________

2. Is the manuscript technically sound, and do the data support the conclusions?

The manuscript must describe a technically sound piece of scientific research with data that supports the conclusions. Experiments must have been conducted rigorously, with appropriate controls, replication, and sample sizes. The conclusions must be drawn appropriately based on the data presented.

Reviewer #1: Yes

Reviewer #2: Yes

________________________________________

3. Has the statistical analysis been performed appropriately and rigorously?

Reviewer #1: Yes

Reviewer #2: Yes

________________________________________

4. Have the authors made all data underlying the findings in their manuscript fully available?

The PLOS Data policy requires authors to make all data underlying the findings described in their manuscript fully available without restriction, with rare exception (please refer to the Data Availability Statement in the manuscript PDF file). The data should be provided as part of the manuscript or its supporting information, or deposited to a public repository. For example, in addition to summary statistics, the data points behind means, medians and variance measures should be available. If there are restrictions on publicly sharing data—e.g. participant privacy or use of data from a third party—those must be specified.

Reviewer #1: Yes

Reviewer #2: Yes

________________________________________

5. Is the manuscript presented in an intelligible fashion and written in standard English?

PLOS ONE does not copyedit accepted manuscripts, so the language in submitted articles must be clear, correct, and unambiguous. Any typographical or grammatical errors should be corrected at revision, so please note any specific errors here.

Reviewer #1: Yes

Reviewer #2: Yes

________________________________________

6. Review Comments to the Author

Please use the space provided to explain your answers to the questions above. You may also include additional comments for the author, including concerns about dual publication, research ethics, or publication ethics. (Please upload your review as an attachment if it exceeds 20,000 characters)

Reviewer #1: (No Response)

Reviewer #2: Now this paper can be accepted for publication because authors did a good revision to the manuscript and the paper is much improved.

________________________________________

7. PLOS authors have the option to publish the peer review history of their article (what does this mean?). If published, this will include your full peer review and any attached files.

If you choose “no”, your identity will remain anonymous but your review may still be made public.

Do you want your identity to be public for this peer review? For information about this choice, including consent withdrawal, please see our Privacy Policy.

Reviewer #1: No

Reviewer #2: Yes: Remus Cretan

________________________________________

Many thanks again for your constructive comments and suggestions, which helped improve the overall quality of our manuscript.

In addition to revising the paper based on the constructive comments and suggestions, we’ve also carefully reviewed the manuscript and made a few other minor revisions where we found necessary.

[NOTE: If reviewer comments were submitted as an attachment file, they will be attached to this email and accessible via the submission site. Please log into your account, locate the manuscript record, and check for the action link "View Attachments". If this link does not appear, there are no attachment files.]

To ensure your figures meet our technical requirements, please review our figure guidelines: https://journals.plos.org/plosone/s/figures

You may also use PLOS’s free figure tool, NAAS, to help you prepare publication quality figures: https://journals.plos.org/plosone/s/figures#loc-tools-for-figure-preparation.

NAAS will assess whether your figures meet our technical requirements by comparing each figure against our figure specifications.

Attachment Submitted filename: Response to editors comments.docx

# Editor report: Decision Letter 2

**DOI:** 10.1371/journal.pone.0350726.r005

**Authors:** Muhammad Khalil Khan

17 Apr 2026

PONE-D-25-50168R2

Representing national images of Self and Others through China’s diplomatic discourse: A corpus-based study

PLOS One

Dear Dr. Li,

Thank you for submitting your manuscript to PLOS ONE. After careful consideration, we feel that it has merit but does not fully meet PLOS ONE’s publication criteria as it currently stands. Therefore, we invite you to submit a revised version of the manuscript that addresses the points raised during the review process.

Please submit your revised manuscript by Jun 01 2026 11:59PM. If you will need more time than this to complete your revisions, please reply to this message or contact the journal office at plosone@plos.org. When you're ready to submit your revision, log on to https://www.editorialmanager.com/pone/ and select the 'Submissions Needing Revision' folder to locate your manuscript file.

Please include the following items when submitting your revised manuscript:

- A letter that responds to each point raised by the academic editor and reviewer(s). You should upload this letter as a separate file labeled 'Response to Reviewers'.
- A marked-up copy of your manuscript that highlights changes made to the original version. You should upload this as a separate file labeled 'Revised Manuscript with Track Changes'.
- An unmarked version of your revised paper without tracked changes. You should upload this as a separate file labeled 'Manuscript'.

If you would like to make changes to your financial disclosure, please include your updated statement in your cover letter. Guidelines for resubmitting your figure files are available below the reviewer comments at the end of this letter.

If applicable, we recommend that you deposit your laboratory protocols in protocols.io to enhance the reproducibility of your results. Protocols.io assigns your protocol its own identifier (DOI) so that it can be cited independently in the future. For instructions see: https://journals.plos.org/plosone/s/submission-guidelines#loc-laboratory-protocols. Additionally, PLOS ONE offers an option for publishing peer-reviewed Lab Protocol articles, which describe protocols hosted on protocols.io. Read more information on sharing protocols at https://plos.org/protocols?utm_medium=editorial-email&utm_source=authorletters&utm_campaign=protocols.

As the corresponding author, your ORCID iD is verified in the submission system and will appear in the published article. PLOS supports the use of ORCID, and we encourage all coauthors to register for an ORCID iD and use it as well. Please encourage your coauthors to verify their ORCID iD within the submission system before final acceptance, as unverified ORCID iDs will not appear in the published article. *Only*  the individual author can complete the verification step; PLOS staff *cannot*  verify ORCID iDs on behalf of authors.

We look forward to receiving your revised manuscript.

Kind regards,

Muhammad Khalil Khan

Academic Editor

PLOS One

Journal Requirements:

If the reviewer comments include a recommendation to cite specific previously published works, please review and evaluate these publications to determine whether they are relevant and should be cited. There is no requirement to cite these works unless the editor has indicated otherwise.

Additional Editor Comments:

Please revise the submission to ensure the language used is neutral and objective in tone and please provide additional references as noted in the journal office requests below and use parentheses for actual quotes. Additionally, I recommend that the author consider using a professional editing service to refine the manuscript's tone and adopt a more neutral approach in the synthesis, discussion, and elaboration sections.

Journal Office requests: During internal review of this submission, PLOS ONE staff editors identified several sentences in the manuscript that may affect its ability to meet the journal’s publication criteria (https://journals.plos.org/plosone/s/criteria-for-publication). To ensure the manuscript maintains a neutral and objective tone, that all statements are appropriately supported by peer-reviewed literature or the presented results, and that claims are not overstated, we recommend revising the text accordingly.

Specifically, it is not clear from the presented results how the following statements are supported by the work. We ask authors to revise the text or provide an appropriate supporting citation.

1. "In contrast, Western countries, particularly the US, seek unilateralism in the name of democracy, freedom, and human rights. They engage in interventions in the internal affairs of other countries and scramble to maintain the hegemonic position and vested interests of developed Western countries in global governance."

2. "Politicians, including president of the US, initiated trade wars and suppressed Chinese enterprises and depicted China as a threat and competitor in their public speeches. In official documents including The National Security Strategy Report, The National Defense Strategy Report, and The Nuclear Posture Review, the Trump administration explicitly labeled China as a “strategic competitor”, declaring that the competition between the US and China is strategic, comprehensive, and long-term"

2a. We also request that links are provided to the above named reports

3. "As part of diplomatic practices, they adopted offensive strategy to criticize the US government and politicians who have wantonly smeared China’s pandemic prevention efforts and projected a negative image of the US."

4. "Through descriptions and evaluations of its unilateralist and imperious behaviors, the spokespersons cast the US in a negative light who is irresponsible domestically for the ineffective pandemic prevention and hegemonic internationally with extended jurisdiction to interfere in other countries’ internal affairs. By contrast, the spokespersons brought to the fore China’s diplomatic principles of multilateralism and win-win cooperation, thereby constructing a favorable image of China as a responsible and cooperative country and highlighting China’s divergence with the West in ideology."

4a. It is unclear that sufficient evidence has been provided in the results themselves to support these statements. As a suggestion, quotes from the spokespersons may provide support.

5. "Their partnership is characterized by frequent high-level exchanges, shared positions on global issues, and a commitment to strengthening cooperation. Notably, both countries face similar external pressures and challenges, leading to a shared commitment to safeguarding their sovereignty and interests"

6. "For instance, the Confucian principle of harmony underscores China’s advocacy for peaceful diplomacy, dialogue, and cooperation rather than confrontation. Simultaneously, righteousness and propriety serve as moral justifications for China’s diplomatic stance and its criticism of perceived injustice or hegemonic behavior in global politics. Such cultural elements enable the MFAC spokespersons to balance assertiveness with courtesy, employing both communicative and offensive discursive strategies strategically. This culturally informed diplomatic rhetoric thus not only helps manage China’s international image but also effectively counters external challenges."

7. "The systematic recurrence of negative evaluative framing in these cases suggests that offensive strategies are not indiscriminately applied but are activated in response to perceived challenges or accusations directed at China"

Conclusions:

8. "On the one hand, they sought to uphold and enhance China’s positive international image by stressing the scientific rigor underlying virus origin investigations and promoting global cooperation in combating COVID-19 as a shared global challenge."

8a. It is not currently clear that this statement is supported by the results presented in the manuscript. We therefore ask the authors to ensure that it is adequately substantiated, either by the study’s findings (e.g., through the inclusion of relevant quotations) or by appropriate references.

9. "On the other hand, they skillfully adopted"

9a. This phrase appears to include subjective language. Please clarify whether the use of the term “skillfully” is justified within the scope of the study. If not directly supported by the results or appropriate references, we recommend revising this phrasing to ensure a more neutral and objective tone.

10. "and defamation attempts spearheaded primarily by the US and some other western media such as BBC."

11. "The analysis shows that national image construction in spokesperson discourse relies on a strategic calibration of cooperative and confrontational strategies, which are selectively deployed in response to geopolitical dynamics and perceived external challenges."

11a. Please provide supportive evidence that the spokesperson discourse was selectively deployed for the stated reasons. If this interpretation is not directly supported by the results or appropriate references, we recommend revising the statement to ensure it accurately reflects the evidence presented.

[Note: HTML markup is below. Please do not edit.]

To ensure your figures meet our technical requirements, please review our figure guidelines: https://journals.plos.org/plosone/s/figures

You may also use PLOS’s free figure tool, NAAS, to help you prepare publication quality figures: https://journals.plos.org/plosone/s/figures#loc-tools-for-figure-preparation.

NAAS will assess whether your figures meet our technical requirements by comparing each figure against our figure specifications.

# Author comment: Author response to Decision Letter 3

**DOI:** 10.1371/journal.pone.0350726.r006

6 May 2026

Journal Office requests: During internal review of this submission, PLOS ONE staff editors identified several sentences in the manuscript that may affect its ability to meet the journal’s publication criteria (https://journals.plos.org/plosone/s/criteria-for-publication). To ensure the manuscript maintains a neutral and objective tone, that all statements are appropriately supported by peer-reviewed literature or the presented results, and that claims are not overstated, we recommend revising the text accordingly.

We thank the editor(s) for drawing our attention to this important issue. We’ve carefully reviewed the manuscript throughout, with particular attention to the Discussion, and Conclusion sections, and revised the relevant passages accordingly.

Specifically, it is not clear from the presented results how the following statements are supported by the work. We ask authors to revise the text or provide an appropriate supporting citation.

1. "In contrast, Western countries, particularly the US, seek unilateralism in the name of democracy, freedom, and human rights. They engage in interventions in the internal affairs of other countries and scramble to maintain the hegemonic position and vested interests of developed Western countries in global governance."

Thanks. We’ve removed “They engage in interventions in the internal affairs of other countries and scramble to maintain the hegemonic position and vested interests of developed Western countries in global governance.” We’ve also reworded them as you can see in the manuscript.

“In contrast, some Western countries, particularly the US, have often articulated critiques of other countries, including China, through discourses of democracy, human rights, and security concerns. For example, the 2017 National Security Strategy described China and Russia as “revisionist powers”. These contextual factors provide a background for interpreting why the US was frequently represented as a salient Other in the MFAC spokespersons’ discourse.”

2. "Politicians, including president of the US, initiated trade wars and suppressed Chinese enterprises and depicted China as a threat and competitor in their public speeches. In official documents including The National Security Strategy Report, The National Defense Strategy Report, and The Nuclear Posture Review, the Trump administration explicitly labeled China as a “strategic competitor”, declaring that the competition between the US and China is strategic, comprehensive, and long-term"

2a. We also request that links are provided to the above named reports

Thanks. We’ve revised this passage to avoid unsupported or politically evaluative wording. We now refer more narrowly to official US strategic documents and provide links to the relevant reports, as requested.

“US official strategic documents during the Trump administration increasingly framed China as a major strategic challenge. For example, the 2018 National Defense Strategy explicitly stated that “China is a strategic competitor”, declaring that the competition between the US and China is strategic, comprehensive, and long-term.

3. "As part of diplomatic practices, they adopted offensive strategy to criticize the US government and politicians who have wantonly smeared China’s pandemic prevention efforts and projected a negative image of the US."

Thanks. We’ve revised the sentence to distinguish between the authors’ analysis and the spokespersons’ own discursive representation. The revised wording now states that the spokespersons represented certain US criticisms as attempts to stigmatize or discredit China, rather than presenting this as the authors’ own judgment.

“As part of these diplomatic practices, the spokespersons used offensive discursive strategies to respond to criticisms from the US government and certain Western media outlets, which were represented in the spokespersons’ discourse as attempts to stigmatize or discredit China’s pandemic response.”

4. "Through descriptions and evaluations of its unilateralist and imperious behaviors, the spokespersons cast the US in a negative light who is irresponsible domestically for the ineffective pandemic prevention and hegemonic internationally with extended jurisdiction to interfere in other countries’ internal affairs. By contrast, the spokespersons brought to the fore China’s diplomatic principles of multilateralism and win-win cooperation, thereby constructing a favorable image of China as a responsible and cooperative country and highlighting China’s divergence with the West in ideology."

4a. It is unclear that sufficient evidence has been provided in the results themselves to support these statements. As a suggestion, quotes from the spokespersons may provide support.

Thanks. We’ve revised the passage so that terms such as “unilateral”, “irresponsible”, and “interference” are clearly presented as evaluative categories found in or inferred from the spokespersons’ discourse, rather than as the authors’ direct characterization of the US. We have also added representative concordance examples to show how negative evaluations of the US were linguistically realized.

“Through recurrent collocates and concordance lines involving terms such as “violate”, “interfere”, and “irresponsible” as can be seen in Figure 1, the spokespersons’ discourse represented the US in negative evaluative terms, particularly in relation to pandemic governance, international responsibility, and intervention in other countries’ affairs. By contrast, China was frequently associated with positively evaluated terms related to cooperation, responsibility, mutual assistance, and multilateralism. These contrastive patterns are consistent with the quantitative distribution reported in Table 4, where China received 3,310 positive representations, whereas the US received 1,400 negative representations.”

Figure 1 Concordance lines of “violate”, “interfere” and “irresponsible” with “US”

5. "Their partnership is characterized by frequent high-level exchanges, shared positions on global issues, and a commitment to strengthening cooperation. Notably, both countries face similar external pressures and challenges, leading to a shared commitment to safeguarding their sovereignty and interests"

Thanks. We’ve revised this statement to avoid overgeneralization but to present China-Russia cooperation as a contextual factor that may help explain the relatively positive representation of Russia in the corpus, rather than as a direct causal claim.

“The relatively positive representation of Russia may be interpreted in relation to the broader context of China-Russia strategic cooperation. In fact, China-Russia relations have in many years involved sustained strategic coordination and that Russia occupies a high-ranking position in China’s partnership diplomacy.”

6. "For instance, the Confucian principle of harmony underscores China’s advocacy for peaceful diplomacy, dialogue, and cooperation rather than confrontation. Simultaneously, righteousness and propriety serve as moral justifications for China’s diplomatic stance and its criticism of perceived injustice or hegemonic behavior in global politics. Such cultural elements enable the MFAC spokespersons to balance assertiveness with courtesy, employing both communicative and offensive discursive strategies strategically. This culturally informed diplomatic rhetoric thus not only helps manage China’s international image but also effectively counters external challenges."

Thanks. We’ve revised this passage to make clear that Confucian concepts are used only as a possible interpretive lens, not as a direct causal explanation for the spokespersons’ discursive strategies. We have also removed wording suggesting that these cultural elements “effectively counter” external challenges.

“Selected elements of traditional Chinese cultural thoughts, such as harmony, righteousness, and propriety, may provide a broader interpretive lens for understanding the coexistence of cooperative and confrontational elements in MFAC spokespersons’ discourse. For example, the Confucian principle of harmony may help contextualize China’s emphasis on peaceful diplomacy, dialogue, and cooperation. At the same time, notions of righteousness and propriety may help explain how criticism of perceived injustice, unilateralism, or hegemonic behavior is morally framed within diplomatic discourse.”

7. "The systematic recurrence of negative evaluative framing in these cases suggests that offensive strategies are not indiscriminately applied but are activated in response to perceived challenges or accusations directed at China"

Thanks. We’ve revised this sentence to avoid making a strong causal claim about strategic intention.

“The concentration of negative evaluative suggests that offensive discursive strategies were not evenly distributed across all external actors. Rather, they were associated primarily with actors as involved in criticism, blame attribution, or perceived challenges to China.”

Conclusions:

8. "On the one hand, they sought to uphold and enhance China’s positive international image by stressing the scientific rigor underlying virus origin investigations and promoting global cooperation in combating COVID-19 as a shared global challenge."

8a. It is not currently clear that this statement is supported by the results presented in the manuscript. We therefore ask the authors to ensure that it is adequately substantiated, either by the study’s findings (e.g., through the inclusion of relevant quotations) or by appropriate references.

Thank you. We’ve revised the conclusion to align it more directly with the corpus findings reported in this study, particularly the positive collocational patterns associated with China. While the spokespersons’ discourse includes cases emphasizing the scientific rigor of virus origin investigations, we’ve reformulated the conclusion to avoid giving undue prominence to claims not directly foregrounded in the Results section.

“On the one hand, the spokespersons’ discourse constructed a positive image of China through communicative strategies, recurrently associating China with cooperation in the global pandemic response.”

9. "On the other hand, they skillfully adopted"

9a. This phrase appears to include subjective language. Please clarify whether the use of the term “skillfully” is justified within the scope of the study. If not directly supported by the results or appropriate references, we recommend revising this phrasing to ensure a more neutral and objective tone.

Thanks. We’ve removed the word “skillfully” to make it more neutral.

“On the other hand, the spokespersons’ discourse also included offensive strategies in response to negative narratives, accusations, and criticisms directed at China primarily by the US and some other western media such as BBC.”

10. "and defamation attempts spearheaded primarily by the US and some other western media such as BBC."

Thanks. We’ve reworded the sentence. “defamation attempts spearheaded” has been removed as you can see from our response to comment 9.

11. "The analysis shows that national image construction in spokesperson discourse relies on a strategic calibration of cooperative and confrontational strategies, which are selectively deployed in response to geopolitical dynamics and perceived external challenges."

11a. Please provide supportive evidence that the spokesperson discourse was selectively deployed for the stated reasons. If this interpretation is not directly supported by the results or appropriate references, we recommend revising the statement to ensure it accurately reflects the evidence presented.

Thanks. We’ve revised the sentence to avoid making a strong claim that is not very directly foregrounded in the Results section. Since the Conclusion section is intended to synthesize the main findings rather than introduce new evidence, we’ve reformulated the statement in more cautious terms.

“The analysis shows that cooperative and offensive discursive strategies used to represent national images in spokesperson discourse were unevenly distributed across the national actors and these observed distributional differences may be interpreted in relation to broader geopolitical dynamics.”

Overall, we have revised the manuscript to ensure that the discussion remains grounded in the corpus results. Where contextual explanations are retained, we’ve reformulated the relevant arguments in more cautious terms and supported them with official documents where appropriate. We’ve also clarified that the analysis concerns representations in the spokespersons’ discourse rather than the authors’ own judgments.

We are very grateful to the editor(s) for these helpful suggestions, which have greatly improved the overall quality of our manuscript.

Attachment Submitted filename: 5.6_Response to Reviewers.docx

# Editor report: Decision Letter 3

**DOI:** 10.1371/journal.pone.0350726.r007

**Authors:** Muhammad Khalil Khan

18 May 2026

Representing national images of Self and Others through China’s diplomatic discourse: A corpus-based study

PONE-D-25-50168R3

Dear Dr. Tao Li

We’re pleased to inform you that your manuscript has been judged scientifically suitable for publication and will be formally accepted for publication once it meets all outstanding technical requirements.

Within one week, you’ll receive an e-mail detailing the required amendments. When these have been addressed, you’ll receive a formal acceptance letter and your manuscript will be scheduled for publication.

An invoice will be generated when your article is formally accepted. Please note, if your institution has a publishing partnership with PLOS and your article meets the relevant criteria, all or part of your publication costs will be covered. Please make sure your user information is up-to-date by logging into Editorial Manager at Editorial Manager® and clicking the ‘Update My Information' link at the top of the page. For questions related to billing, please contact billing support.

If your institution or institutions have a press office, please notify them about your upcoming paper to help maximize its impact. If they’ll be preparing press materials, please inform our press team as soon as possible -- no later than 48 hours after receiving the formal acceptance. Your manuscript will remain under strict press embargo until 2 pm Eastern Time on the date of publication. For more information, please contact onepress@plos.org.

Kind regards,

Muhammad Khalil Khan

Academic Editor

PLOS One

Additional Editor Comments (optional):

Reviewers' comments:

# Editor report: Acceptance letter

**DOI:** 10.1371/journal.pone.0350726.r008

**Authors:** Muhammad Khalil Khan

PONE-D-25-50168R3

PLOS One

Dear Dr. Li,

I'm pleased to inform you that your manuscript has been deemed suitable for publication in PLOS One. Congratulations! Your manuscript is now being handed over to our production team.

At this stage, our production department will prepare your paper for publication. This includes ensuring the following:

* All references, tables, and figures are properly cited

* All relevant supporting information is included in the manuscript submission,

* There are no issues that prevent the paper from being properly typeset

You will receive further instructions from the production team, including instructions on how to review your proof when it is ready. Please keep in mind that we are working through a large volume of accepted articles, so please give us a few days to review your paper and let you know the next and final steps.

Lastly, if your institution or institutions have a press office, please let them know about your upcoming paper now to help maximize its impact. If they'll be preparing press materials, please inform our press team within the next 48 hours. Your manuscript will remain under strict press embargo until 2 pm Eastern Time on the date of publication. For more information, please contact onepress@plos.org.

You will receive an invoice from PLOS for your publication fee after your manuscript has reached the completed accept phase. If you receive an email requesting payment before acceptance or for any other service, this may be a phishing scheme. Learn how to identify phishing emails and protect your accounts at https://explore.plos.org/phishing.

If we can help with anything else, please email us at customercare@plos.org.

Thank you for submitting your work to PLOS ONE and supporting open access.

Kind regards,

PLOS ONE Editorial Office Staff

on behalf of

Dr. Muhammad Khalil Khan

Academic Editor

PLOS One
